# Conjugate Polymer Anchor Enhancing Matrix Vacuum Stability and Improving MALDI MSI via Ion Bond

**DOI:** 10.1002/advs.202406296

**Published:** 2024-07-17

**Authors:** Xi Yu, Junyu Chen, Zhengzhou Li, Duo Shen, Huihui Liu, Zongxiu Nie

**Affiliations:** ^1^ Beijing National Laboratory for Molecular Sciences Key Laboratory of Analytical Chemistry for Living Biosystems Institute of Chemistry Chinese Academy of Sciences Beijing 100190 China; ^2^ University of Chinese Academy of Sciences Beijing 100190 China

**Keywords:** conjugate polymer anchor, MALDI MSI, matrix, vacuum stability

## Abstract

Poor vacuum stability limits the application of many matrices in matrix‐assisted laser desorption/ionization mass spectrometry imaging (MALDI MSI) that requires long‐term measurement duration in high vacuum. In this study, a new approach using conjugate polymer anchor to protect unstable matrix from volatilizing in MALDI source based on ion bond is provided. Unlike strong covalent bonds which often introduce unnecessary groups, the weaker ion bonds are more conducive to breaking under laser radiation while effectively preventing matrix volatilization in a vacuum environment. The results confirm that conjugate polymer anchor will neither introduce additional ion peaks nor affect signal intensity, yet maintains comparable quantification properties. Vacuum stability of three kinds of typical matrices is enhanced using polymer anchors, and the in situ MALDI MS imaging of mouse brain and liver cancer is improved significantly.

## Graphical abstract

Vacuum stability is important for matrix in matrix‐assisted laser desorption/ionization mass spectrometry imaging (MALDI MSI) requiring long term in high vacuum. Ion bond is used to protect unstable matrix rather than covalent bond, which is strong and difficult to break for MALDI ion source. Combined with corresponding acid/base conjugate pair, matrix is able to keep stability in vacuum and be released in source under the laser irradiation.

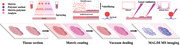

## 1 Introduction

Matrix‐assisted laser desorption/ionization mass spectrometry (MALDI MS) and MALDI MS imaging (MSI) are widely used analytical methods in clinical, biological, and environmental research.^[^ ^1^, ^2^, ^3^ ^]^ In high‐vacuum MALDI source, matrix absorbs laser energy and helps analyte in gasifying and ionizing.^[^ ^4^, ^5^, ^6^ ^]^ Thus, the vacuum stability of matrix is important for high‐throughput MALDI MS measurement, especially for MALDI MSI which requires longer measurement duration.^[^ ^7^ ^]^ To enhance matrix vacuum stability, scientists have developed many approaches in recent years, such as employing covalent bonds to tether matrix with to involatile molecules or polymers, as well as developing new matrices.^[^ ^8^ ^]^ Although these methods do prevent matrix from volatilizing in vacuum environment, they may inadvertently alter matrix‐assisted properties, such as diminishing ion signal intensity and introducing extraneous background peaks.^[^ ^9^, ^10^, ^11^, ^22^ ^]^

Unlike covalent bonds, ion bonds as a kind of intermolecular force rely on the electrostatic interaction between positive and negative ion, making them more easily dissociated with external force. In particular, most of small organic molecule matrices containing functional groups like amido, carboxyl, and phenolic hydroxyl groups can donate or accept proton to enhance the analyte ionization.^[^ ^12^, ^13^, ^14^, ^15^, ^16^, ^17^ ^]^ By paring with corresponding acid/base conjugates, matrices are able to maintain stability in vacuum and release in source upon laser irradiation, provided the acid/base ratio is appropriately controlled. Furthermore, to enhance vacuum stability and avoid producing extra matrix signal peaks, polymers with high molecular weights and inert chemistry properties were utilized instead of small molecules. Adjusting the degree of polymerization helps resolve the signal overlap of the noise and the analyte, ensuring effective matrix protection.

In this work, we demonstrate a new approach based on ion binding to prevent instable matrix from volatilizing in vacuum. Alkaline matrix (or acidic matrix) could combine with the corresponding polymer acid (or polymer base) and become a conjugate acid–base pair, which can protect matrix in vacuum environment like anchor anchored boat in storm. We named this approach as conjugate polymer anchor. MALDI matrix such as 1, 8‐bis(dimethyl amino)naphthalene (DMAN)^[^ ^18^ ^]^ featuring in analyzing lipids are volatile under vacuum environment, restricting its application in MS imaging. After simply mixing DMAN with polyacrylic acid (PAA), the conjugate acid–base pair of DMAN‐PAA would be prepared, and this makes DMAN become vacuum‐stable after being grappled by PAA. Furthermore, polymers would not introduce background signals in the small molecule mass range and the ionization efficiency of small molecules was also improved using DMAN‐PAA. From the results, we found the ion bond between matrix and polymer anchor is able to protect DMAN in MALDI source under high vacuum and be broken by laser irradiation during ionization process, facilitating its application in MALDI MSI. In addition, more useful vacuum‐unstable matrices, including the dual‐mode ion mode reactive matrix 2‐hydrazinoquinoline (2‐HQ),^[^ ^19^ ^]^ and 2‐nitrophloroglucinol (2‐NPG)^[^ ^20^ ^]^ which can produce multiply charged ions for proteins, were also evaluated to investigate the universality of conjugate polymer anchor for enhancing matrix vacuum stability.

## 2 Results and Discussion

### 2.1 Conjugate Polymer Anchor Enhancing DMAN Vacuum Stability and Improving Mouse Brain Imaging

**Figure 1:**
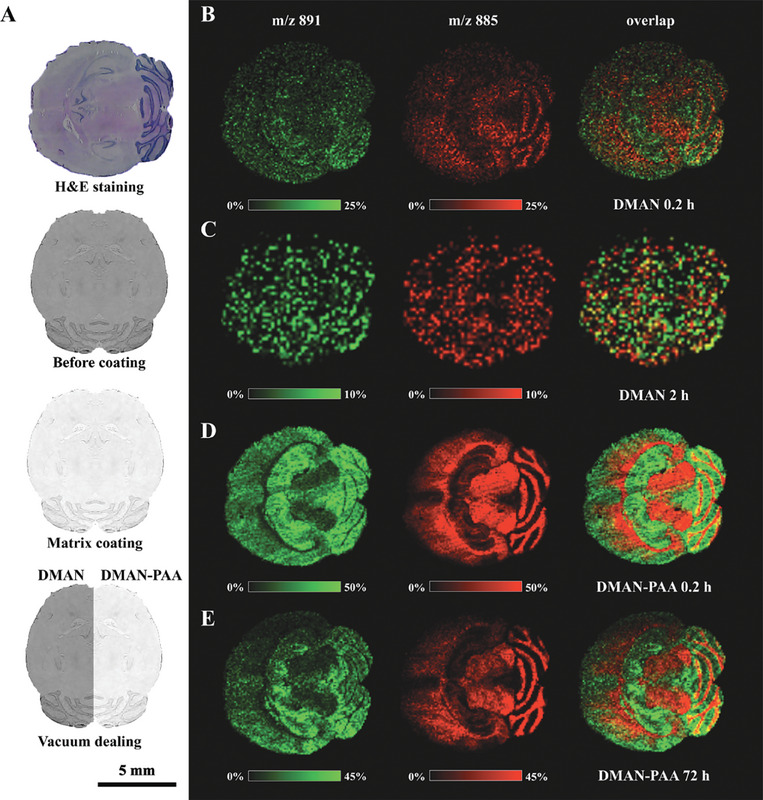
Conjugate polymer anchor enhancing DMAN vacuum stability and improving mouse brain imaging. A) H&E staining of mouse brain horizontal section and schematic of PAA protecting matrix in vacuum. MALDI MSI consequences of DMAN‐assisted imaging after B) 0.2 h and C) 2 h vacuum dealing, and DMAN‐PAA‐assisted imaging after D) 0.2 h and E) 72 h. MS imaging experiments were performed in negative mode with laser energy adjusted to 55% and the special resolution set at 100 µm.

DMAN, a widely used MALDI MS matrix, with two tertiary amines which was effective for fatty acid analysis but not suitable for MSI due to vacuum instability, was used in the present study. To address this problem, a simple yet effective approach was developed by conjugating polymer anchor using polymerized acid PAA with DMAN via the ionic bond, which was easily fragmented by MALDI laser. The tissue section after coating with matrix binding to conjugate polymer anchor was directly subjected to MALDI MSI. The imaging result using individual DMAN and DMAN binding to PAA (DMAN‐PAA) over different time periods under vacuum were compared with same experimental conditions.^[^ ^21^ ^]^ The tissue slices with matrices coating were measured after exposed in the MALDI ion source of high vacuum for 0.2 h as control. The DMAN‐assisted LDI MSI of mouse brain horizontal section lacked clarity due to vacuum instability (Figure S14, Supporting Information), hindering the differentiation of encephalic regions based on the spatial distribution of metabolites or lipids, as shown in **Figure** **1**b. And the available information was even worse for the section exposed for 2 h as shown in the Figure 1c, which meant almost all DMAN volatilized in vacuum environment. Thus, DMAN was not suitable to use in MALDI MSI due to poor vacuum stability. The optical images and optical microscope images of DMAN and DMAN‐PAA before and after vacuum dealing are shown in Figures S12 and S13 (Supporting Information). The matrix coated on the tissue slices would not volatilize immediately even when all preparation work readied until the sample were exposed in the ion source of MALDI for certain term to uniform the vacuum dealing time. The use of DMAN‐PAA facilitated much clearer spatial distribution images of various metabolites and lipids in mouse brain sections with same 100 µm spatial resolution. Two representative phosphatides exhibiting complementary spatial distribution were highlighted as shown in Figure 1d. The encephalic region from the imaging results was consistent with the results of hematoxylin and eosin (H&E) staining (Figure 1d). Even after being exposed in vacuum for 24 h, the ion signal intensity using DMAN‐PAA was almost not decreased (Figure S24, Supporting Information). It demonstrated that conjugate polymer anchor could protect matrix, enhance its vacuum stability and improve the MSI. The UV–vis absorption spectra are shown in Figure S1 (Supporting Information). The ^1^H NMR spectra of DMAN, PAA, and DMAN‐PAA are shown in Figures S2,S3,S8,S10, and S11 (Supporting Information).

### 2.2 Conjugate Polymer Anchor Enhancing DMAN Vacuum Stability and Improving In Situ Hepatocellular Carcinoma Imaging

**Figure 2:**
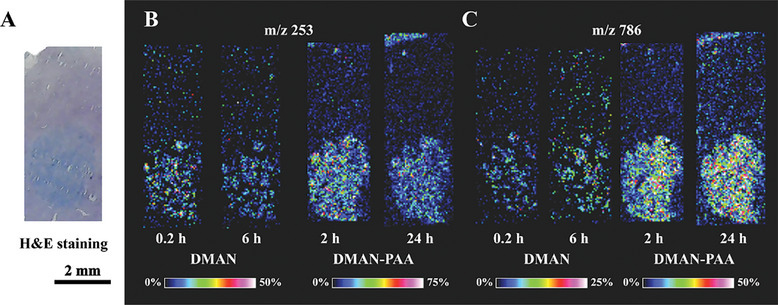
Conjugate polymer anchor enhancing DMAN vacuum stability and improving in situ hepatocellular carcinoma imaging. A) H&E staining of liver tissue section. B,C) MALDI MSI consequences of DMAN assisted imaging after 0.2 and 6 h vacuum dealing, and DMAN‐PAA‐assisted imaging after 2 and 24 h. MS imaging experiments were performed in negative mode with laser energy adjusted to 55% and the special resolution set at 50 µm.

Furthermore, the practical application of conjugate polymer anchor was also taken into discussion. The DMAN‐PAA‐assisted LDI MSI of liver section with in situ hepatocellular carcinoma was compared with the results with DMAN. A comparable imaging area, encompassing both the tumor and adjacent normal tissue was examined. Metabolites and lipids with significant difference between liver cancer tumor and normal tissue were able to be identified, in which the MSI consequences of two representative lipids with same experimental conditions was shown in **Figure** **2**. The results showed that DMAN‐PAA‐assisted imaging distinctly delineated the tumor region from the surrounding tissue (Figure 2d), whereas the utilization of DMAN alone rendered ambiguous outcomes (Figure 2b), comparing with H&E staining results (Figure 2a). Notably, even after 24 h of exposure in the MALDI ion source, DMAN‐PAA could still maintain consistent imaging outcomes with enough distinguishability, whereas DMAN exhibited poor performance. It suggested that conjugate polymer anchor was effective in protecting vacuum unstable matrix and improving imaging.

### 2.3 Standard Analytes Measuring, Calculable Quantification, and Vacuum Stability of DMAN and DMAN‐PAA

**Figure 3:**
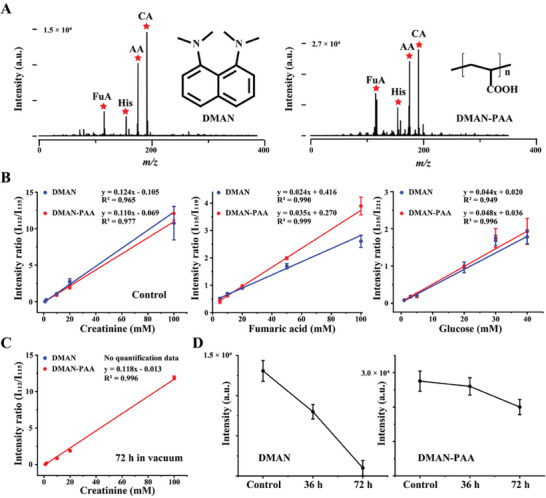
Standard analytes measuring, calculable quantification and vacuum stability of DMAN and DMAN‐PAA. A) Representative MALDI MS spectra of DMAN and DMAN‐PAA, respectively, assisted four standard samples, histidine (His), ascorbic acid (AA), citric acid (CA), and fumaric acid (FuA). B) Linearity curves of DMAN and DMAN‐PAA, respectively, assisted creatinine, fumaric acid, and glucose. C) Linearity curves of creatinine after 72 h vacuum dealing. D) Ion signal intensity of DMAN and DMAN‐PAA, respectively, assisted CA after 0.2, 36, and 72 h vacuum dealing. MS imaging experiments were performed in negative mode with laser energy adjusted to 55%.

To ensure the laser's ability to cleave the ionic bond between conjugate polymer anchor and the matrix, and to confirm that the polymer will not interfere with the matrix's function, DMAN and DMAN‐PAA‐assisted LDI MS analysis of standard analytes was performed. Four analytes, namely histidine (His), ascorbic acid (AA), citric acid (CA), and fumaric acid (FuA), were chosen (Figures S17,S19, and S20, Supporting Information). Their mass spectra are shown in **Figure** **3**a, revealing similar ion signals and intensities for both DMAN and DMAN‐PAA. In addition, the quantifiability of the matrix was assessed by evaluating the ion signal intensity ratios of glucose to ^13^C6‐glucose (I215/I221), creatinine to D3‐creatinine (I112/I115), and fumaric acid to ^13^C4‐fumaric acid (I115/I119). Remarkably, both DMAN and DMAN‐PAA exhibited comparable linearity curves within the same concentration range (Figure 3b). Furthermore, the stability of the matrix and its quantification properties were examined after 72 h of vacuum exposure. DMAN‐PAA demonstrated well‐preserved ion signal intensities for standard analytes and maintained consistent linearity curves (Figure 3c). In contrast, DMAN exhibited some ion signals with lower intensity and significant deviation (Figure 3d). This indicates that the polymer anchor does not impede matrix quantification and effectively preserves its stability under vacuum conditions.

### 2.4 PAH Enhanced 2‐NPG Vacuum Stability and Improved Mouse Brain Imaging

**Figure 4:**
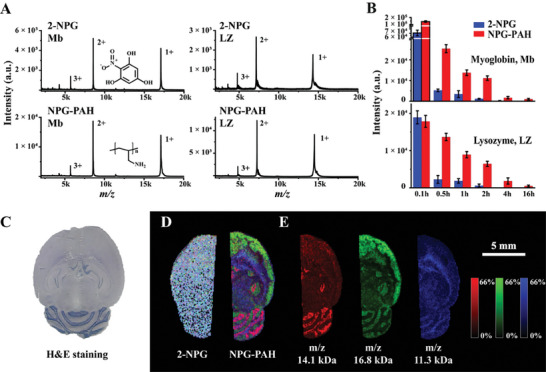
PAH enhancing 2‐NPG vacuum stability improved mouse brain imaging. A) Representative MALDI MS spectra of 2‐NPG and NPG‐PAH, respectively, assisted two standard proteins, myoglobin (Mb) and lysozyme (LZ) after 0.5 h vacuum dealing. B) Ion signal intensity of 2‐NPG and NPG‐PAH, respectively, assisted Mb and LZ with double positive charges (2+). C) H&E staining of mouse brain horizontal section. D) Overlap of three proteins from MALDI MSI consequences of 2‐NPG and NPG‐PAH assisted imaging, and E) individual proteins imaging consequences. MS imaging experiments were performed in positive mode with laser energy adjusted to 60% and the special resolution set at 100 µm.

2‐nitrophloroglucinol (2‐NPG), a matrix with three phenolic hydroxyl groups, facilitated the generation of multiple charges peaks for high molecular weight proteins. However, its vulnerability to vacuum instability restricted its application in MS imaging. To evaluate the universality of conjugate polymer anchor, conjugate polymer poly(allylamine hydrochloride) (PAH) with amines was selected to show the protection for 2‐NPG under the vacuum environment. Due to the terrible vacuum instability, there was a notable discrepancy in ion signal intensity between different measuring times, especially at earlier intervals. Thus, individual 2‐NPG and NPG binding to PAH (NPG‐PAH) assisted LDI MS analysis of myoglobin (Mb) and lysozyme (LZ) were conducted following a 6‐min vacuum exposure as a control. Compared with individual 2‐NPG, the signal intensities of Mb and LZ using NPG‐PAH were higher but exhibited a decline over time (**Figure** **4**a). This suggested that PAH could help 2‐NPG to keep enough ion signal intensity during the first 2 h (Figure 4b). So the imaging duration of horizontal sections of mouse brain was all controlled within 2 h with 100 µm spatial resolution. The overlay figures of three representative proteins using 2‐NPG and NPG‐PAH‐assisted LDI MSI are shown in Figure 4d,e. The spatial distribution of proteins with PAH‐protected matrix was clearer, while there was almost no ion information available from 2‐NPG‐assisted LDI MS imaging (Figures S16 and S18, Supporting Information). Although PAH can enhance the vacuum stability of 2‐NPG and improve mouse brain imaging effectively, NPG‐PAH keep enough ion signal intensity only within 2 h (Figure S21, Supporting Information). It was proposed that the phenolic hydroxyl group on 2‐NPG was difficult to give H^+^ due to poor ionization constant. In fact, we had tried to use polyethyleneimine (PEI) with much stronger alkalinity than PAH to improve the ionization efficiency of 2‐NPG, but it did not work well. The 1H NMR spectra of 2‐NPG, PAH, and NPG‐PAH are shown in Figures S6,S7, and S9 (Supporting Information).

### 2.5 PAA Enhancing 2‐HQ Vacuum Stability Improved Mouse Brain and In Situ Liver Cancer Tissue Imaging

**Figure 5:**
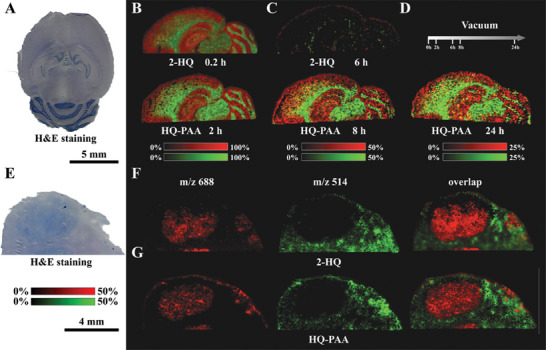
PAA enhancing 2‐HQ vacuum stability and improved mouse brain and in situ liver cancer tissue imaging. A) H&E staining of mouse brain horizontal section. Overlap of two features from MALDI MSI consequences of 2‐HQ‐assisted imaging after B) 0.2 h and C) 6 h and HQ‐PAA‐assisted imaging after B) 2 h, C) 6 h, and D) 24 h. E) H&E staining of in situ hepatocellular carcinoma section. MALDI MSI consequences of F) 2‐HQ and G) HQ‐PAA‐assisted in situ liver cancer tissue imaging. MS imaging experiments were performed in negative mode with laser energy adjusted to 55% and the special resolution set at 100 µm for mouse brain and 50 µm for liver.

In addition, 2‐HQ, a dual ion mode reactive matrix with one primary amine and one secondary amine, was also explored to assess the broad applicability of conjugate polymer anchor. Initially, individual 2‐HQ and 2‐HQ binding to PAA (HQ‐PAA) assisted LDI MSI of mouse brain horizontal section on one ITO glass slide with 100 µm spatial resolution were carried out. Metabolites and lipids including phosphatides with unique distribution in tissue were able to be evaluated. Two lipids with complementary spatial distributions in brain section were selected and their overlay images were shown in **Figure** **5**. Notably, 2‐HQ demonstrated suitability for use in MALDI MSI, as the encephalic region appeared clear even without considering vacuum instability (Figure S15, Supporting Information). Similarly, HQ‐PAA provided comparable results even after 2 h in the ion source (Figure 5b). However, the imaging result using 2‐HQ deteriorated significantly after just 6 h of vacuum exposure, displaying faint contours (Figure 5c). In contrast, HQ‐PAA maintained excellent ion signal intensity with prolonged processing time, lasting up to 24 h (Figure 5d). Furthermore, the 2‐HQ and HQ‐PAA assisted LDI MSI of liver section with in‐situ hepatocellular carcinoma were evaluated (Figures S22 and S23, Supporting Information). The significant boundary between tumor region and tissue adjacent to cancer was found from the imaging consequences of two representative lipids with 50 µm spatial resolution (Figure 5f,g). Specially, we also found several additional metabolites and lipids from the imaging results of HQ‐PAA‐assisted LDI MS analysis of liver section after 2 and 14 h of vacuum exposure, and the distribution were consistent with H&E staining. These findings collectively suggested that conjugate polymer anchor could enhance vacuum stability and improve MS imaging.

### 2.6 Conjugate Polymer Anchor Enhanced Unstable Matrix via Ion Bond

**Figure 6:**
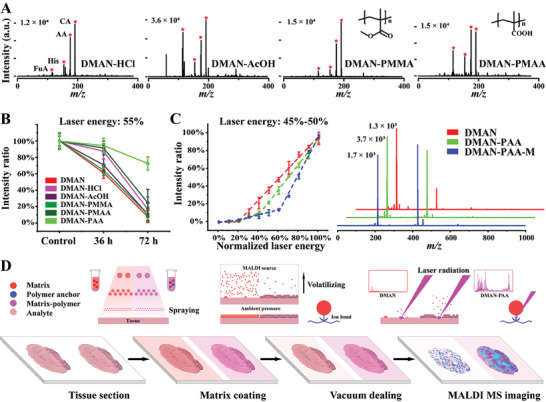
Conjugate polymer anchor enhanced unstable matrix via ion bond. A) Representative MALDI MS spectra of DMAN‐assisted four standard samples, His, AA, CA, and FuA with four kinds of additives, HCl, AcOH, PMMA, and PMAA. B) Ion signal intensity ratio of matrices assisted CA with different term vacuum dealing. C) Ion signal intensity ratio of matrices with normalized increasing laser energy for DMAN, DMAN‐PAA, and DMAN‐PAA with large concentration PAA and representative MALDI MS spectra. D) Schematic diagram of conjugate polymer anchor enhancing matrix vacuum stability and improving MALDI MSI via ion bond, which prevents matrix molecule volatilizing in vacuum and releases matrix under laser radiation. MS imaging experiments were performed in negative mode with laser energy adjusted from 45% to 50%.

Lastly, we inferred the protection mechanism of conjugate polymer anchor based on ion bond, including ion bond preventing unstable matrix from volatilizing in MALDI source with high vacuum and releasing this kind of constraint under laser radiation. The former was confirmed using small molecule weight acids, namely hydrochloric acid (HCl) and acetic acid (AcOH), and similar polymers, namely poly(methacrylic acid) (PMAA) with carboxy group but poorer acidity and poly(methyl methacrylate) (PMMA) without carboxy group to enhance DMAN vacuum stability (**Figure** **6**a,b). It was found that conjugate acid could protect matrix with amido and the effect of improving vacuum stability depended on the acidity strength and its own stability of acid. The latter was checked via evaluating the ion signal intensity of matrix mixed with different concentration of conjugate polymer anchor under increasing laser energy to show the ion bond breaking happen after absorbing a little but enough energy (Figure 6c). Obviously, there was inflection point on the curve of intensity to energy for DMAN‐PAA, which was affected by the rate of PAA, while DMAN provided linear curve. It suggested that ion bond was weaker than covalent bond but enough bond strength could prevent matrix molecule from volatilizing like anchor in MALDI ion source with high vacuum and release matrix when using laser radiation to desorb, ionize and detect analyte (Figure 6d). The 1H NMR spectra of 2‐HQ and HQ‐PAA are shown in Figures S4 and S5 (Supporting Information).

## 3 Conclusion

We successfully provided a new approach using conjugate polymer anchor to prevent instable matrix from volatilizing in vacuum based on ion bond. The experiment suggested that DMAN unable to keep stable in MALDI source could work well for MSI with the protection of PAA. We also confirmed the universality of conjugate polymer anchor during evaluating the effectivity of PAH and PAA for helping instable 2‐NPG and 2‐HQ not to volatilize, respectively. Furthermore, we made sure the practical application of conjugate polymer anchor from the imaging of mouse brain section to in situ liver cancer tumor section. Finally, we revealed that matrix linked to conjugate polymer anchor which was broken under laser in the process of MALDI MSI can enhance matrix vacuum stability and improving imaging. In conclusion, compared with previous reported methods, the conjugate polymer anchors could be commercially available with low price. They could enhance the vacuum stability of matrix that is easily volatilized in vacuum without increasing background interference and compromising sensitivity. This approach based on ion bond improved the application of typical matrices and provide valuable insights for the development of new matrices in MSI applications.

## Conflict of Interest

The authors declare no conflict of interest.

## Supporting information

Supporting Information

## Data Availability

The data that support the findings of this study are available in the supplementary material of this article.
