# Supplementary material for: Conjugate Polymer Anchor Enhancing Matrix Vacuum Stability and Improving MALDI MSI via Ion Bond
Source: Adv Sci (Weinh). 2024 Jul 17;11(35):2406296. doi: 10.1002/advs.202406296 (PMC11425218; doi:10.1002/advs.202406296)
Supplement: Supplementary file 1 — Supporting Information [file ADVS-11-2406296-s001.docx]

**Conjugate Polymer Anchor Enhancing Matrix Vacuum Stability Improving MALDI MSI via Ion Bond**

Xi Yu,^[a,b]^ Junyu Chen,^[a,b]^ Zhengzhou Li,^[a,b]^ Duo Shen,^[a,b]^ Huihui Liu,*^[a,b]^ Zongxiu Nie*^[a,b]^

[a] X. Yu, J. Chen, Z. Li, D. Shen, H. Liu, Z. Nie
Beijing National Laboratory for Molecular Sciences, Key Laboratory of Analytical Chemistry for Living Biosystems
Institute of Chemistry, Chinese Academy of Sciences
Beijing 100190, China
E-mail: [znie@iccas.ac.cn](mailto:znie@iccas.ac.cn); [hhliu@iccas.ac.cn](mailto:hhliu@iccas.ac.cn)

[b] X. Yu, J. Chen, Z. Li, D. Shen, H. Liu, Z. Nie
University of Chinese
Academy of Sciences
Beijing 100190, China

**Contents**

[**Methods** 4](#_Toc167801289)

[Chemical and Reagents. 4](#_Toc167801290)

[Preparation of matrix. 4](#_Toc167801291)

[Characterizations. 4](#_Toc167801292)

[Preparation of standard sample. 5](#_Toc167801293)

[MALDI-MS for standard sample analysis. 5](#_Toc167801294)

[Animal Tumor Model. 5](#_Toc167801295)

[Preparation of tissue slices. 5](#_Toc167801296)

[Preparation of matrix coating tissue slices. 5](#_Toc167801297)

[MALDI MS and MALDI MSI. 5](#_Toc167801298)

[Vacuum dealing. 6](#_Toc167801299)

[Data acquisition and analysis. 6](#_Toc167801300)

[**Characterizations** 7](#_Toc167801301)

[Figure S1. UV-Vis absorption spectra. 7](#_Toc167801302)

[Figure S2. 1H NMR spectra of DMAN. 8](#_Toc167801303)

[Figure S3. 1H NMR spectra of DMAN-PAA. 9](#_Toc167801304)

[Figure S4. 1H NMR spectra of 2-HQ. 10](#_Toc167801305)

[Figure S5. 1H NMR spectra of HQ-PAA 11](#_Toc167801306)

[Figure S6. 1H NMR spectra of 2-NPG. 12](#_Toc167801307)

[Figure S7. 1H NMR spectra of NPG-PAH. 13](#_Toc167801308)

[Figure S8. 1H NMR spectra of PAA. 14](#_Toc167801309)

[Figure S9. 1H NMR spectra of PAH. 15](#_Toc167801310)

[Figure S10. 1H NMR spectra of DMAN and DMAN-PAA with different concentration. 16](#_Toc167801311)

[Figure S11. 1H NMR spectra of DMAN and DMAN-PAA with different concentration (chemical shift from 8.5 to 6.5 ppm). 17](#_Toc167801312)

[Figure S12. Optical images of 2-NPG, 2-HQ, NPG-PAH, and HQ-PAA before and after vacuum dealing. 18](#_Toc167801313)

[Figure S13. Optical microscope images of 2-NPG, 2-HQ, NPG-PAH, and HQ-PAA before and after vacuum dealing. 19](#_Toc167801314)

[**MALDI MS spectra** 20](#_Toc167801315)

[Figure S14. Mass spectra of MALDI MSI (DMAN) 20](#_Toc167801316)

[Figure S15. Mass spectra of MALDI MSI (2-HQ). 22](#_Toc167801317)

[Figure S16. Mass spectra of MALDI MSI (2-NPG). 23](#_Toc167801318)

[Figure S17. Mass spectra of calculable quantification for DMAN and DMAN-PAA assisted fumaric acid. 24](#_Toc167801319)

[Figure S18. Mass spectra of 2-NPG and NPG-PAH assisted Mb and LZ with different vacuum dealing. 25](#_Toc167801320)

[Figure S19. Mass spectra of DMAN-PAA assisted four standard samples with different concentration of PAA. 26](#_Toc167801321)

[Figure S20. Changing ion signal intensity of DMAN-PAA assisted CA before and after vacuum dealing. 27](#_Toc167801322)

[**MALDI MS imaging** 28](#_Toc167801323)

[Figure S21. PAH enhancing 2-NPG vacuum stability improved mouse brain coronal section imaging. 28](#_Toc167801324)

[Figure S22. Imaging consequences of 2-HQ assisted LDI MS with RP mode. 29](#_Toc167801325)

[Figure S23. MALDI MS imaging consequences of PAA improving 2-HQ imaging property besides vacuum stability. 30](#_Toc167801326)

[Figure S24. MALDI MS imaging consequences of PAA improving DMAN imaging property (24 h vacuum dealing). 31](#_Toc167801327)

# Methods

Chemical and Reagents. Asparagine, histidine (His), ascorbic acid (AA), citric acid (CA), fumaric acid (FuA), creatinine, glucose, 1,8-bis(dimethylamino)naphthalene (DMAN), and 2-nitrophloroglucinol (2-NPG) were purchased from Sigma-Aldrich (St. Louis, MO, USA). ^13^C_4_-fumaric acid, methyl-D_3_-creatinine, and ^13^C_6_-glucose were purchased from Aladdin Co., Ltd. (Shanghai, China). Polyacrylic acid (PAA), 2-hydrazinoquinoline (2-HQ), poly(methacrylic acid) (PMAA), and poly(methyl methacrylate) (PMMA) were purchased from TCI Development Co., Ltd. (Shanghai, China). Poly(allylamine hydrochloride) (PAH) were purchased from J&K Scientific Co., Ltd. (Beijing, China). Myoglobin (Mb) and lysozyme (LZ) were purchased from Sinopharm Chemical Reagent Co., Ltd. (Shanghai, China). Male Kunming mice (6 weeks old, 20−23 g) were provided by the Vital River Company (Beijing, China). All animal experiments were approved by the Animal Care and Use Committee of the Chinese Academy of Sciences and were carried out according to the NIH Guide for the Care and Use of Laboratory Animals (National Institutes of Health Publication, No. 3040-2, revised 1999, Bethesda, MD).

Preparation of matrix. Individual matrices DMAN and 2-NPG were dissolved in ACN/H_2_O (7/3, v/v) solution at the concentration of 10 mg mL^-1^. Individual 2-HQ was dissolved in MeOH/H_2_O (7/3, v/v) solution at the concentration of 10 mg mL^-1^. Conjugate polymer anchor protecting matrices were prepared by mixing the individual matrices additional 1 mg mL^-1^ PAA and 10 mg mL^-1^ poly(allylamine hydrochloride) dissolved in the same solvent, respectively. Other matrices were prepared by mixing 0.7 mg mL^-1^ hydrochloric acid, 1.1 mg mL^-1^ acetic acid, 1.6 mg mL^-1^ PMAA and 1.8 mg mL^-1^ PMMA with 10 mg mL^-1^ DMAN, respectively. For optimizing concentration, matrices were prepared by mixing 5, 2.5, 1.5, 1, 0.6, 0.3, and 0.1 mg mL^-1^ PAA and 10 mg mL^-1^ DMAN, respectively. Notably, the concentration of matrix for MALDI MSI was the half for MALDI MS.

Characterizations. Ultraviolet–visible (UV-Vis) absorption spectra were performed on PE Lambda 1050+. DMAN (0.01 mg/mL), 2-NPG (0.01 mg/mL), 2-HQ (0.01 mg/mL), PAA (0.001 mg/mL), PAH (0.01 mg/mL), DMAN-PAA (0.01 mg/mL DMAN, 0.001 mg/mL PAA), NPG-PAH (0.01 mg/mL 2-NPG, 0.01 mg/mL PAH), and HQ-PAA (0.01 mg/mL 2-HQ, 0.001 mg/mL PAA) with 70% ACN (ACN:H_2_O, v/v, 7:3) as solvent were prepared for UV-Vis. PAA and PAH shown basically no absorption (200 – 800 nm), and there was no obvious different between DMAN and DMAN-PAA, between 2-HQ and HQ-PAA, and between 2-NPG and NPG-PAH. Proton nuclear magnetic resonance (^1^H NMR) spectra were performed on Bruker AV III 400 HD (400 MHz). DMAN (10 mg/mL), 2-NPG (10 mg/mL), 2-HQ (10 mg/mL), PAA (1 mg/mL), PAH (10 mg/mL), DMAN-PAA (10 mg/mL DMAN, 1 mg/mL PAA), NPG-PAH (10 mg/mL 2-NPG, 10 mg/mL PAH), and HQ-PAA (10 mg/mL 2-HQ, 1 mg/mL PAA) with 70% CD_3_CN (CD_3_CN:D_2_O, v/v, 7:3) as solvent were prepared for ^1^H NMR. DMAN, δ = 7.34 – 7.21 (d, 2H), δ = 7.34 – 7.21 (t, 2H), 6.92 (d, 2H), 2.71 (s, 12H). DMAN-PAA, δ = 7.84 – 7.26 (m, 4H), 7.58 - 6.90 (m, 2H), 2.99 - 2.60 (d, 12H), 2.15 – 1.95 (m, 0.1H), 1.65 – 1.25 (m, 0.2H). 2-HQ, δ = 7.89 (m, 1H), 7.62 (m, 2H), 7.52 (m, 1H), 7.23 (m, 1H), 6.85 (m, 1H). HQ-PAA, δ = 7.80 (m, 1H), 7.53 (m, 2H), 7.47 (m, 1H), 7.18 (m, 1H), 6.79 (m, 1H), 2.22 (m, 0.1H), 1.59 (t, 0.2H). 2-NPG, δ = 6.07 (s, 2H). NPG-PAH, δ = 6.01 (s, 2H), 2.96 (m, 2H), 2.51 – 1.52 (m, 1H) 1.43 (m, 2H). PAA, δ = 2.47 – 2.13 (m, 1H), 1.67 (t, 2H). PAH, δ =2.95 (m, 2H), 2.53 – 1.50 (m, 1H) 1.41 (m, 2H). Specially, there was crosslink network between PAA and DMAN with multifunctional groups, so the ^1^H NMR spectrum of DMAN-PAA was different with DMAN due to space steric hindrance effect, in which DMAN-PAA show more peaks. For individual DMAN, there were two double peaks (b and d) and one triple peaks (c) of naphthalene ring (chemical shift from 7.4 – 6.8 ppm), in which one double peaks and one triple peaks (c and d) overlapped, while DMAN-PAA with low concentration and poor space steric hindrance effect became double: four double peaks (b, b’, d and d’) and two triple peaks (c and c’) of naphthalene ring (chemical shift from 8.0 – 6.8 ppm), in which one double peaks and one triple peaks (c and d) also overlapped. When increasing the concentration of DMAN and PAA, two double peaks (b’ and d’) gradually overlapped for strong space steric hindrance effect. Finally, DMAN-PAA (10 mg/mL DMAN, 1 mg/mL PAA) showed four multiple peaks of naphthalene ring.

Preparation of standard sample. A mixture (5 × 10^-3^ M) consisting of His, AA, CA and FuA was prepared by being dissolved in deionized water. Proteins Mb and LZ were prepared at the concentration of 5 mg mL^-1^. For fumaric acid and creatinine quantitation, ^13^C_4_-fumaric acid and methyl-D_3_-creatinine were prepared at the concentration of 20 mM, and the concentration ratios of fumaric acid/^13^C_4_-fumaric acid were 5, 1, 0.5, 0.25 and 0.125, respectively, and the concentration ratios of creatinine/methyl-D_3_-creatinine were 5, 2.5, 1, 0.5 and 0.25, respectively. For glucose quantitation, ^13^C_6_-glucose was prepared at the concentration of 10 mM, and the concentration ratios of creatinine/methyl-D_3_-creatinine were 1, 2, 5, 20, 30, and 40 mM, respectively.

MALDI-MS for standard sample analysis. Normally, standard samples (1 μL) were mixed with equal volume matrices (1 μL). And ^13^C_4_-fumaric acid (1 μL) and fumaric acid (1 μL) with different concentration ratio were mixed with equal volume matrices (2 μL) for quantitation. Similarly, the quantitation of creatinine was prepared with same condition. The obtained mixture was deposited on the indium tin oxide coated glass slides, drying at room temperature for MALDI-MS analysis.

Animal Tumor Model. The orthotopic H22 liver tumor model was established according to the following steps: The BALB/c mice were anesthetized by inhalation gas with isoflurane through the R500IP small animal anesthesia machine (RWD Life Science Co., Ltd, Shenzhen, Guangdong, China), and the body temperature of the animals was maintained at 37 ± 1 °C throughout the whole surgery using a heating pad. After a longitudinal cut was made in the abdomen and the liver was exposed, 1×10^6^ H22 cells (dispersed in 50 μL normal saline solution) mixed with matrigel at a volume ratio of 1:1 were directly injected into the left lobe of the liver with a micro syringe. The surgical incisions were sutured using 6-0 absorbable surgical sutures (Jinhuan Medical, Shanghai, China).

Preparation of tissue slices. Freshly frozen mouse brain organs were cut into sections with 10 μm thickness by a Leica CM1950 cryostat (Leica Microsystems GmbH, Wetzlar, Germany) at -20 °C, and mounted onto the indium tin oxide coated glass slides. Before matrix coating, the horizontal sections of mouse brain and the liver tissue sections for DMAN, 2-HQ, DMAN-PAA and HQ-PAA were placed into a vacuum desiccator and dried for half an hour. The horizontal sections and coronal sections of mouse brain for 2-NPG and NPG-PAH coating were pretreated with EtOH/H_2_O (7/3, v/v) for 30 s, EtOH for 30 s, EtOH for 30 s, EtOH/CH_3_/AcOH (6/3/1, v/v/v) for 30 s, TFA (0.2% in H_2_O) for 30 s and H_2_O for 5 min in sequence and dried at room temperature.

Preparation of matrix coating tissue slices. Matrix coating by SoniCoat were with a flow rate of solution at 70 μL/min, an ultrasonic power of 0.8 W, a flow rate of supply gas at 0.9 L/min, a nozzle moving velocity of 5.0 mm/s, a line spacing of 1.5 mm, a nozzle height of 60 mm and cover of 3 layers for DMAN, 2-NPG, DMAN-PAA and NPG-PAH. For 2-HQ and HQ-PAA, the moving velocity was 3.5 mm/s and the cover layer was 5. And the specific spraying area was adjusted according to the full size of tissue sections.^21^

MALDI MS and MALDI MSI. MALDI MS and MSI experiments were performed on an Ultraflextreme MALDI-TOF MS (Bruker Daltonics, Billerica, MA) equipped with a 355 nm smart beam Nd:YAG pulsed laser. The vacuum pressure of MALDI source was 4.0 × 10^-6^ mbar. The laser spot size was set to “small”.

For DMAN, 2-HQ, DMAN-PAA and HQ-PAA assisted LDI MS analysis and imaging performed in the negative reflection mode, the voltage of ion sources 1 and 2 were set as 20.00 and 17.70 kV, respectively. The reflection voltage was set as 21.10 kV. Unless other specified, the laser repetition rate was set at 1000 Hz and the laser energy was adjusted to 55%. Each spectrum consisted of 200 shots over the mass range of 0 ~ 1000 Da.

For measuring the matrix signal of DMAN with increasing laser energy, MALDI MS was performed in the negative reflection mode with laser energy from 45% to 50% and each spectrum consisted of 100 shots.

For 2-HQ and HQ-PAA assisted LDI MS analysis and imaging performed in the positive reflection mode, the voltage of ion sources 1 and 2 were set as 20.00 and 17.70 kV, respectively. The reflection voltage was set as 21.10 kV. Unless other specified, the laser repetition rate was set at 1000 Hz and the laser energy was adjusted to 55%. Each spectrum consisted of 200 shots over the mass range of 0 ~ 1000 Da.

For 2-NPG and NPG-PAH assisted MALDI MS analysis and MSI, all experiments was performed in the positive linearity mode. The voltage of ion sources 1 and 2 were set as 20.00 and 17.85 kV, respectively. The reflection voltage was set as 21.10 kV. The laser repetition rate was set at 1000 Hz and laser energy was adjusted to 60%. Each spectrum consisted of 1000 shots over the mass range of 2000 ~ 22000 Da.

Vacuum dealing. Matrix coating tissue slices were place into MALDI source with high vacuum (4.0 × 10^-6^ mbar) for vacuum dealing before MALDI MS imaging. The clock started once the sample was successfully injected. The tissue slices with matrices coating were measured after 0.2 h vacuum dealing for control group to uniform the waiting time.

Data acquisition and analysis. The data of MALDI MS from at least three experiments were expressed as means ± standard deviation, and all independent experiments were performed at least three times. The peak detection, extraction, and normalization were performed in flexAnalysis (version 3.4), and the signal to noise were over 3 based on snap peak detection algorithm. The acquired data of MALDI MSI were processed by flexImaging (version 3.0) with the total ion current (TIC) normalization.

# Characterizations


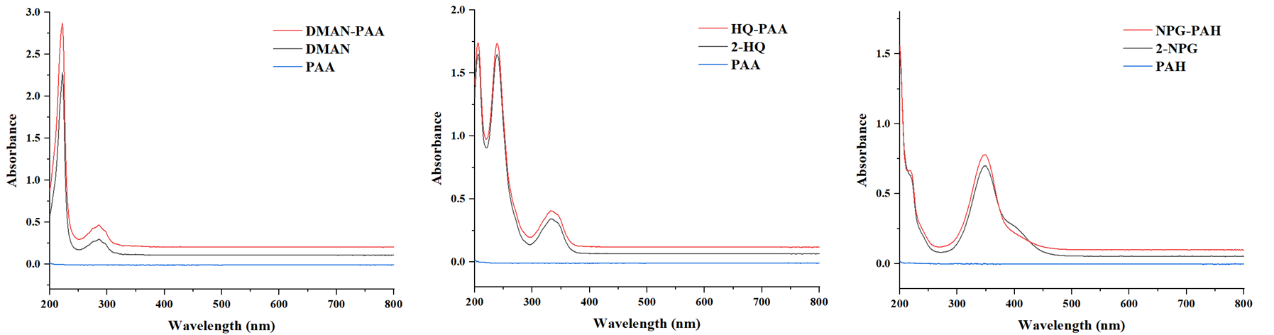


Figure S1. UV-Vis absorption spectra of DMAN-PAA (DMAN 0.01 mg/mL, PAA 0.001 mg/mL), DMAN (0.01 mg/mL), PAA (0.001 mg/mL), HQ-PAA (2-HQ 0.01 mg/mL, PAA 0.001 mg/mL), 2-HQ (0.01 mg/mL), NPG-PAH (NPG 0.01 mg/mL, PAH 0.01 mg/mL), 2-NPG (0.01 mg/mL), PAH (0.01 mg/mL) in 70% ACN (ACN:H_2_O, v/v, 7:3).


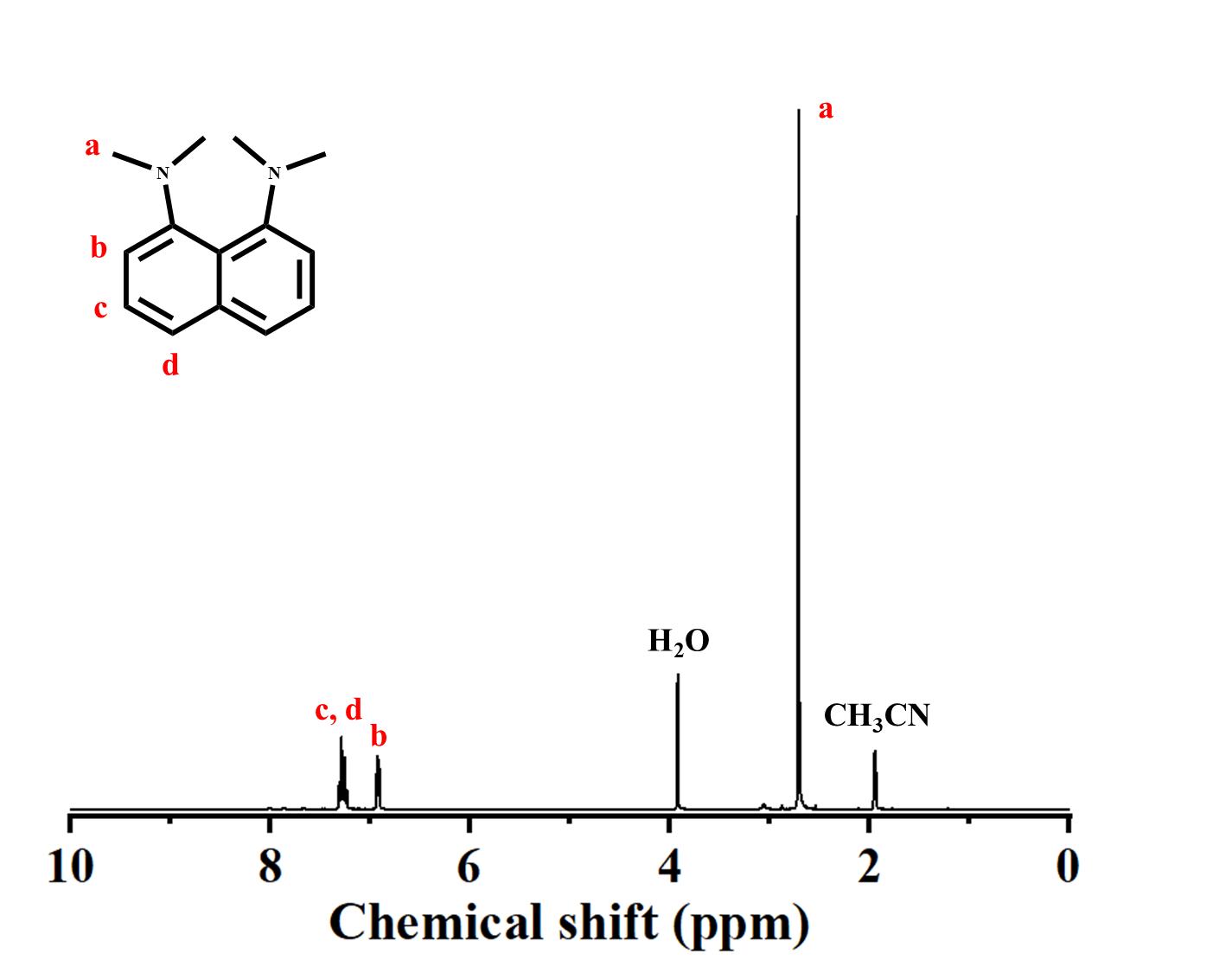


Figure S2. ^1^H NMR spectrum of DMAN. ^1^H NMR (400 MHz), δ = 7.34 – 7.21 (d, 2H), δ = 7.34 – 7.21 (t, 2H), 6.92 (d, 2H), 2.71 (s, 12H).


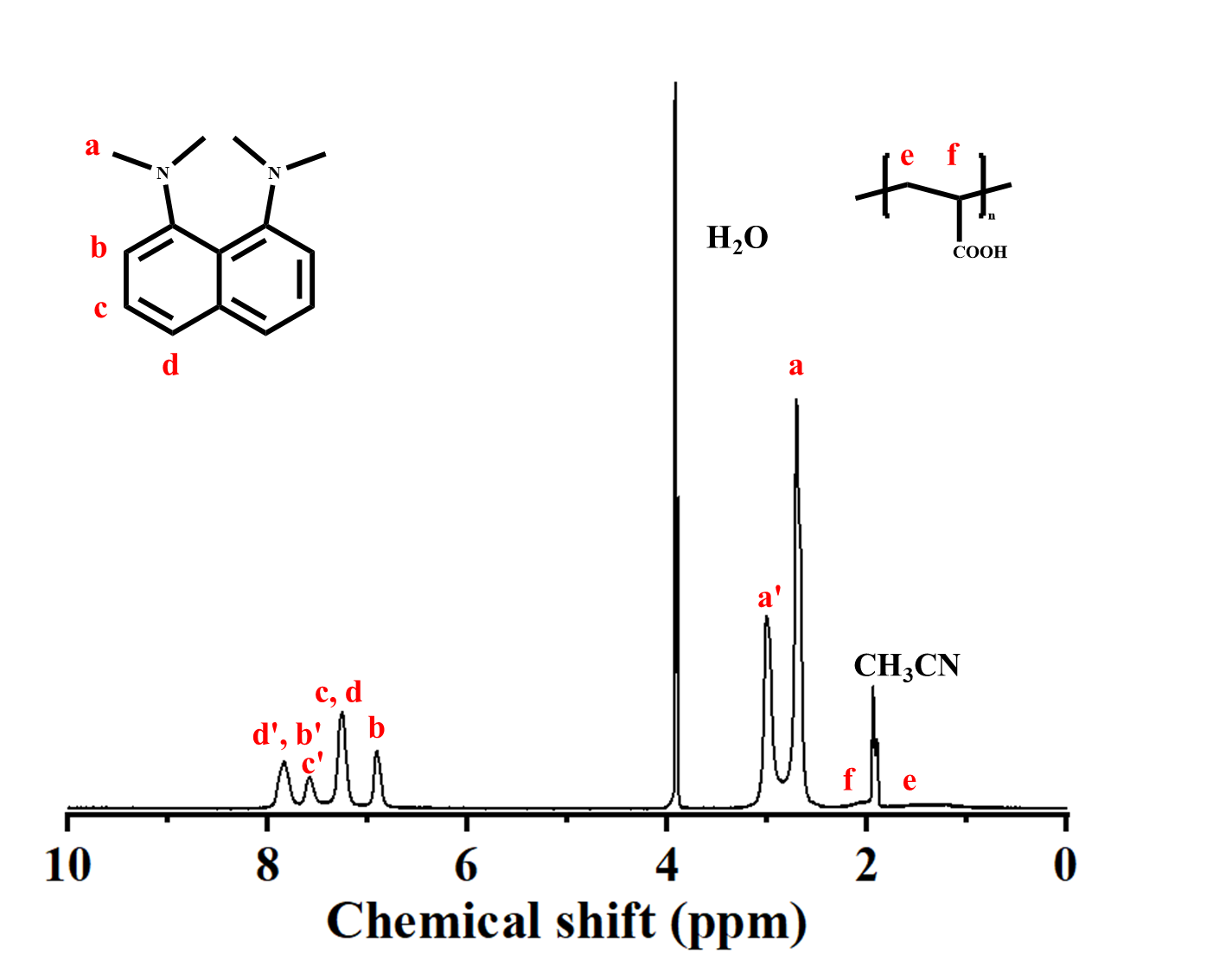


Figure S3. ^1^H NMR spectrum of DMAN-PAA. ^1^H NMR (400 MHz), δ = 7.84 – 7.16 (m, 2H), 7.84 – 6.90 (m, 2H), 7.68 – 7.16 (m, 2H), 2.99 - 2.60 (d, 12H), 2.15 – 1.95 (m, 0.1H), 1.65 – 1.25 (m, 0.2H).


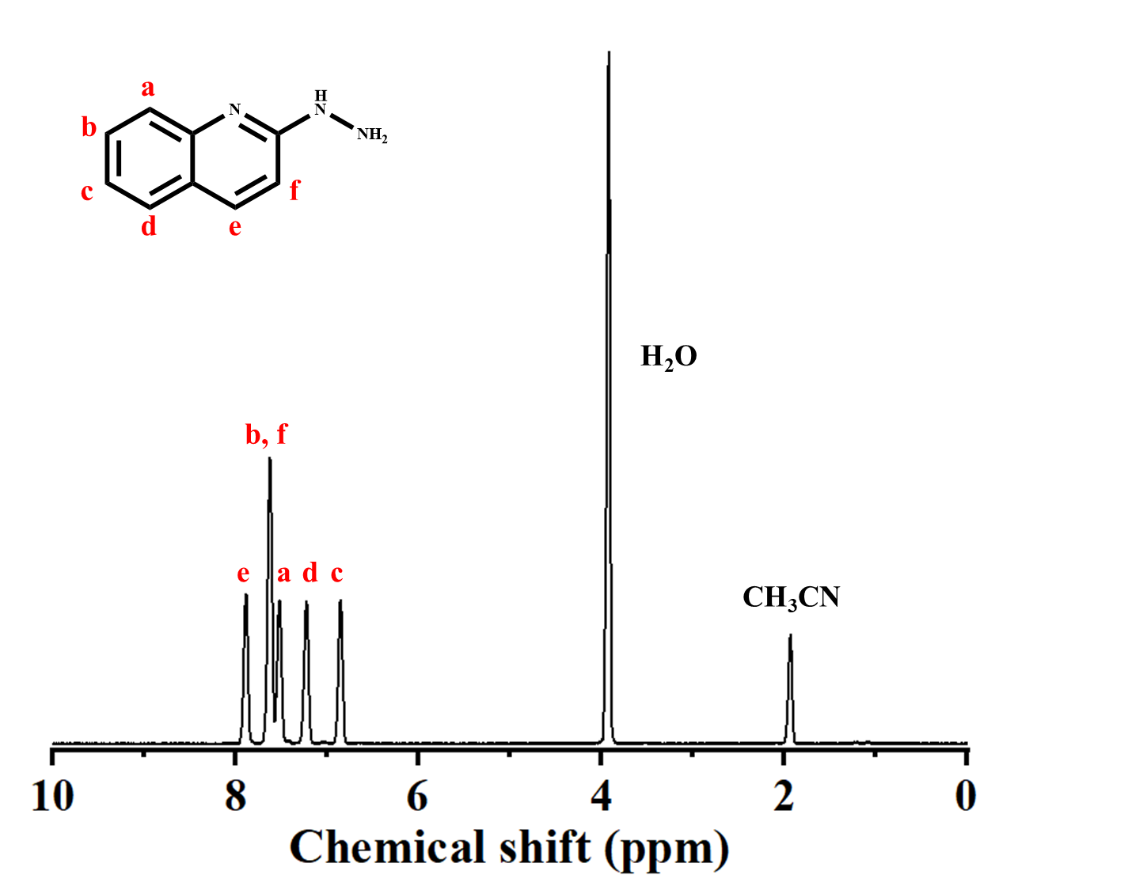


Figure S4. ^1^H NMR spectrum of 2-HQ. ^1^H NMR (400 MHz), δ = 7.89 (m, 1H), 7.62 (m, 2H), 7.52 (m, 1H), 7.23 (m, 1H), 6.85 (m, 1H).


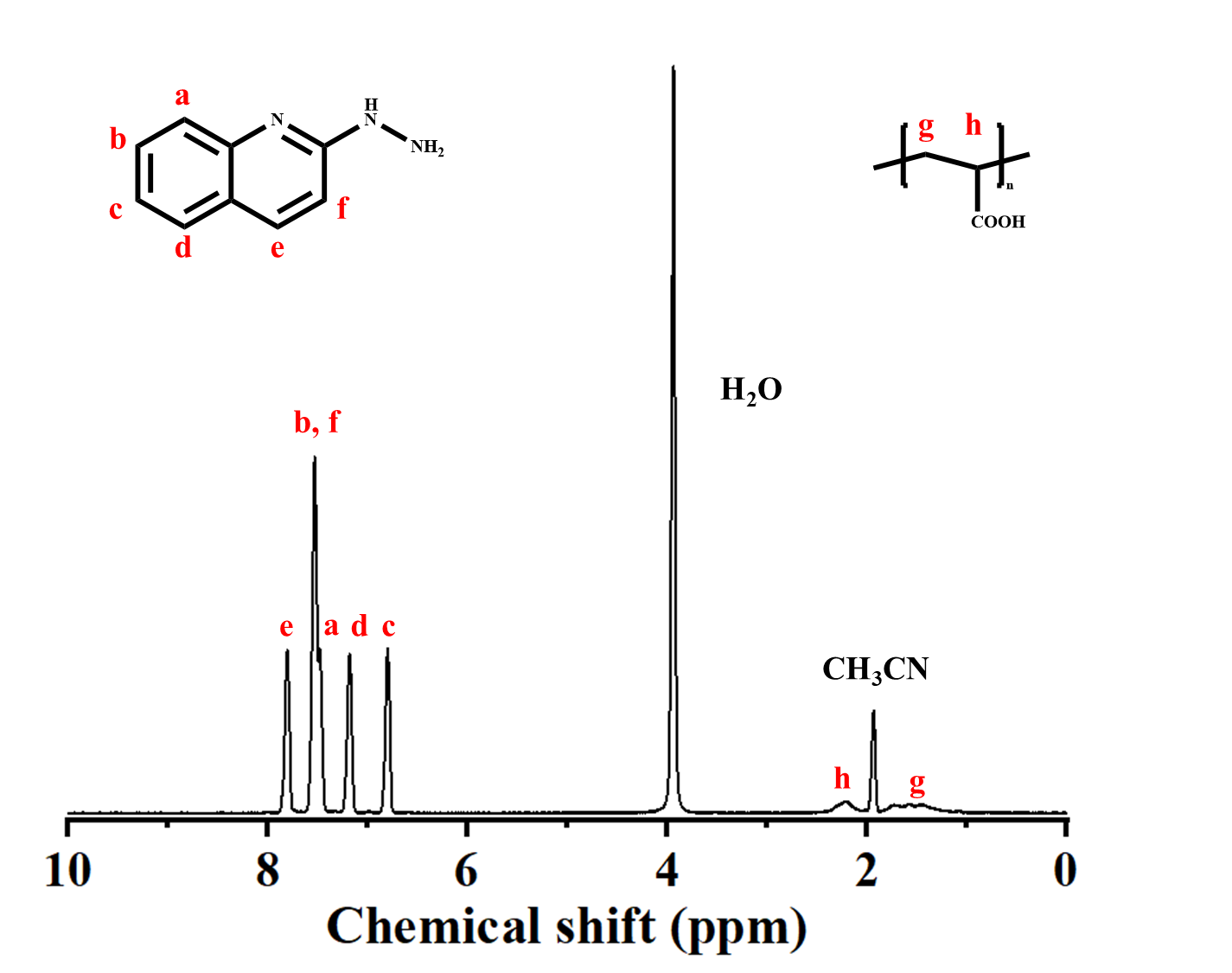


Figure S5. ^1^H NMR spectrum of HQ-PAA. ^1^H NMR (400 MHz), δ = 7.80 (m, 1H), 7.53 (m, 2H), 7.47 (m, 1H), 7.18 (m, 1H), 6.79 (m, 1H), 2.22 (m, 0.1H), 1.59 (t, 0.2H).


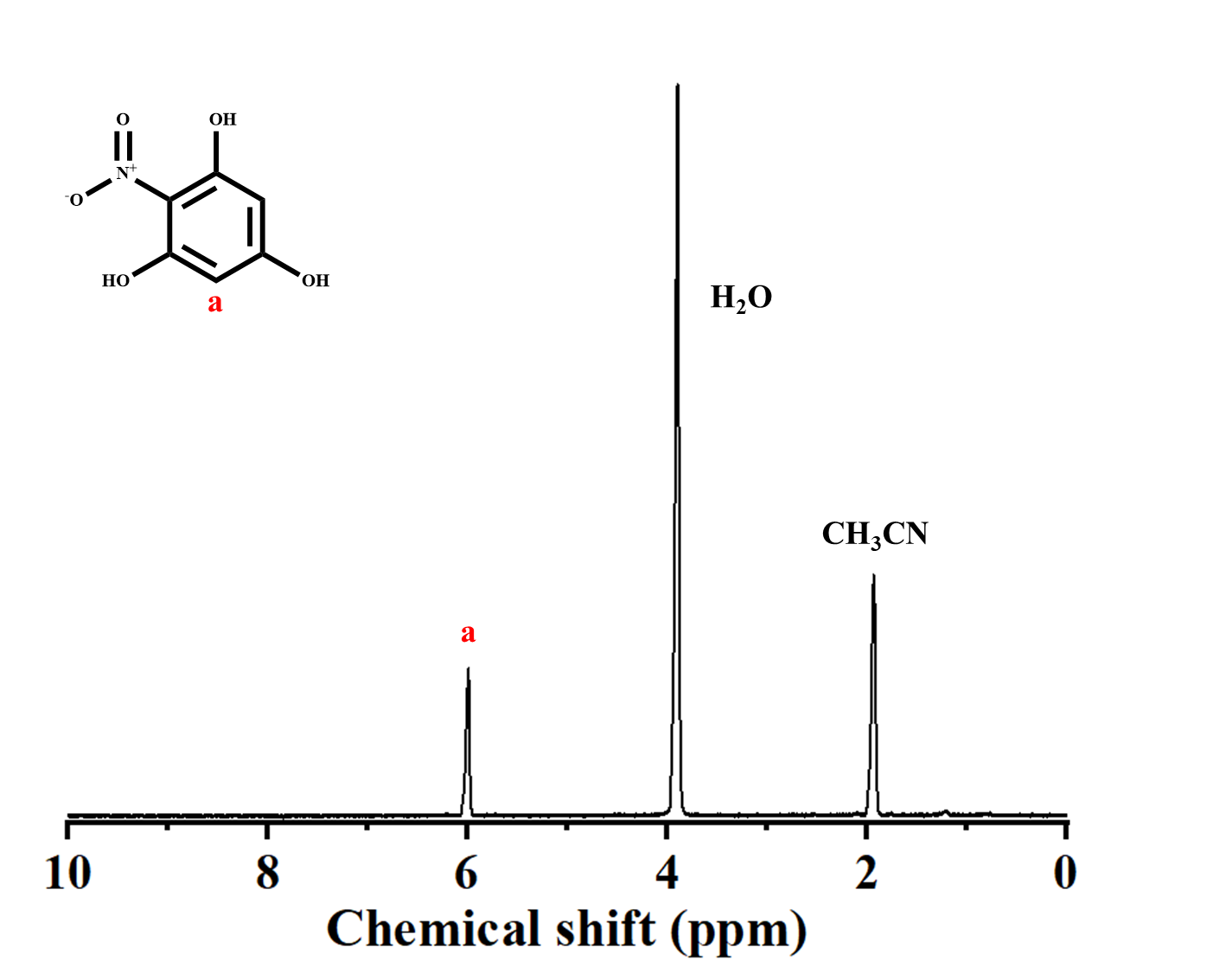


Figure S6. ^1^H NMR spectrum of 2-NPG. ^1^H NMR (400 MHz), δ = 6.07 (s, 2H).


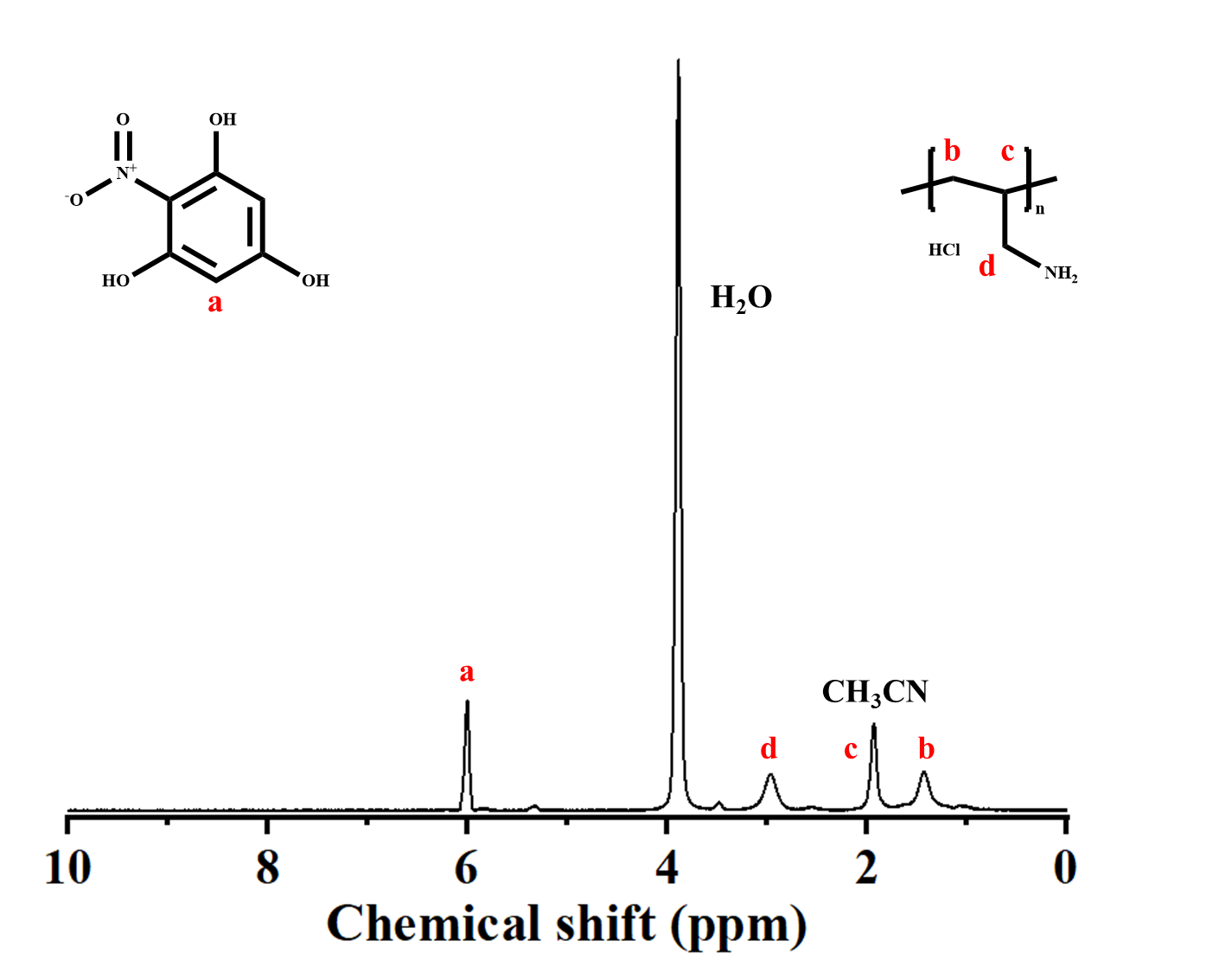


Figure S7. ^1^H NMR spectrum of NPG-PAH. ^1^H NMR (400 MHz), δ = 6.01 (s, 2H), 2.96 (m, 2H), 2.51 – 1.52 (m, 1H) 1.43 (m, 2H).


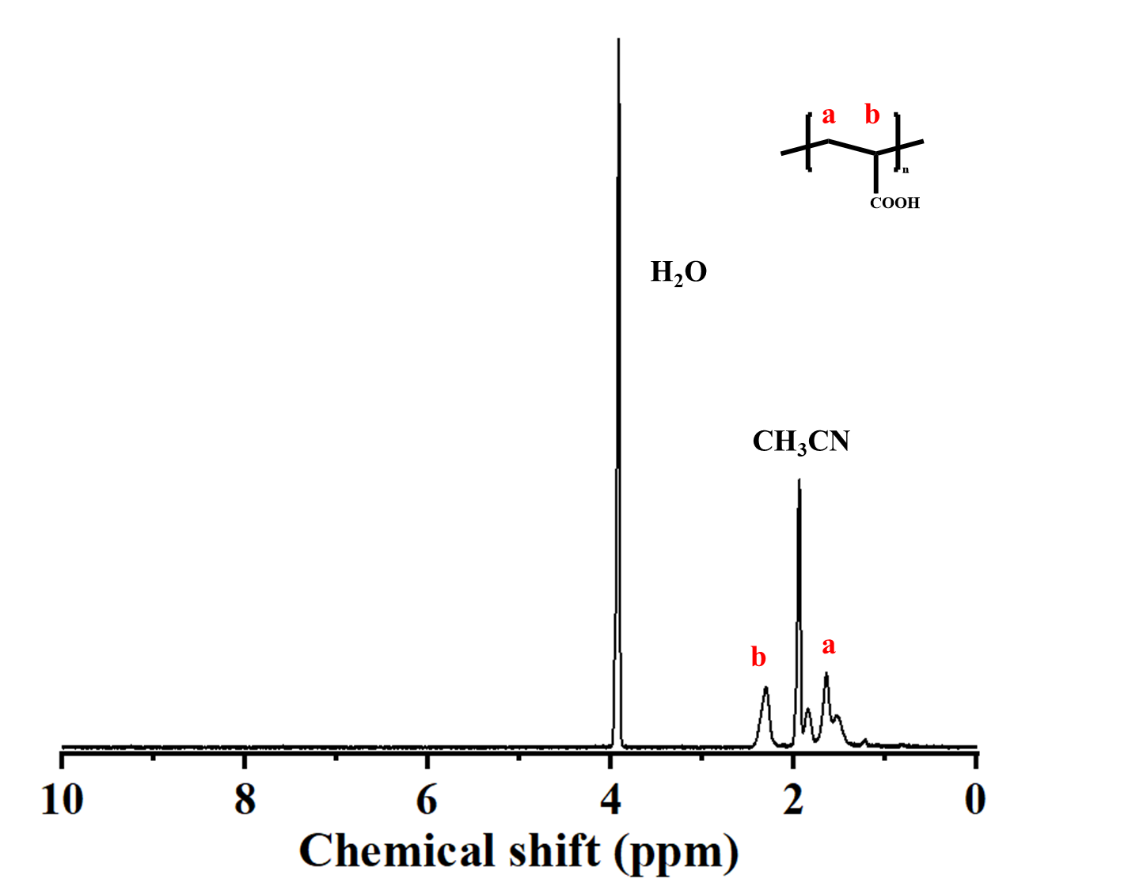


Figure S8. ^1^H NMR spectrum of PAA. ^1^H NMR (400 MHz), δ = 2.47 – 2.13 (m, 1H), 1.67 (t, 2H).


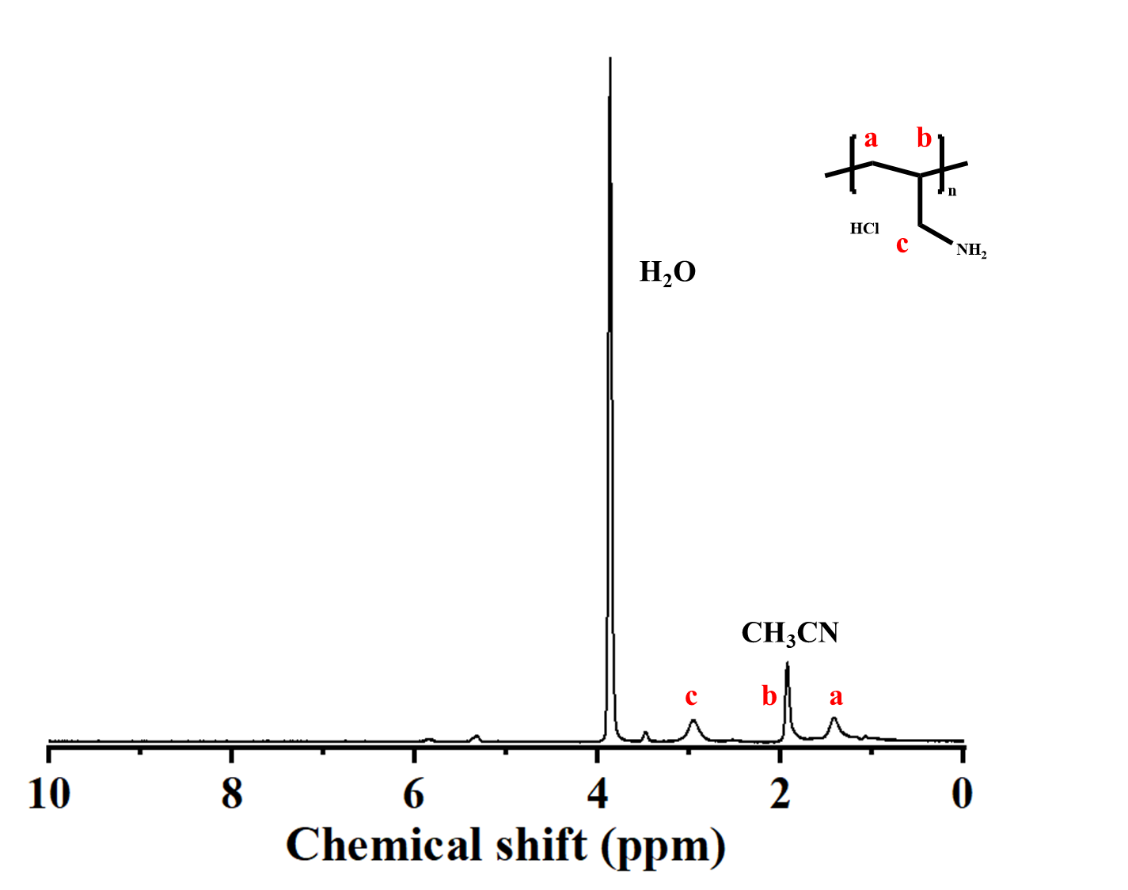


Figure S9. ^1^H NMR spectrum of PAH. ^1^H NMR (400 MHz), δ =2.95 (m, 2H), 2.53 – 1.50 (m, 1H) 1.41 (m, 2H).


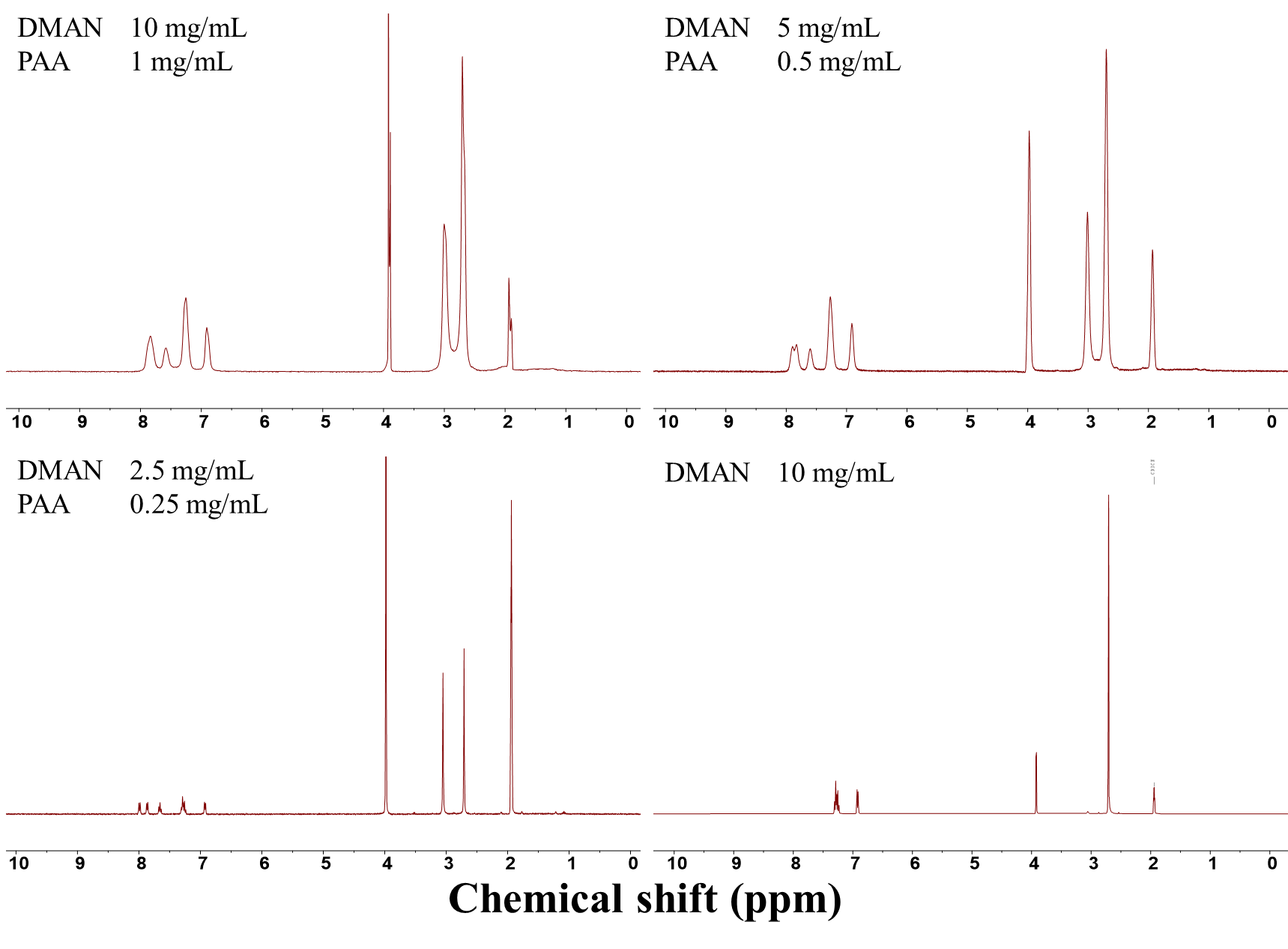


Figure S10. ^1^H NMR spectra of DMAN and DMAN-PAA with different concentration.


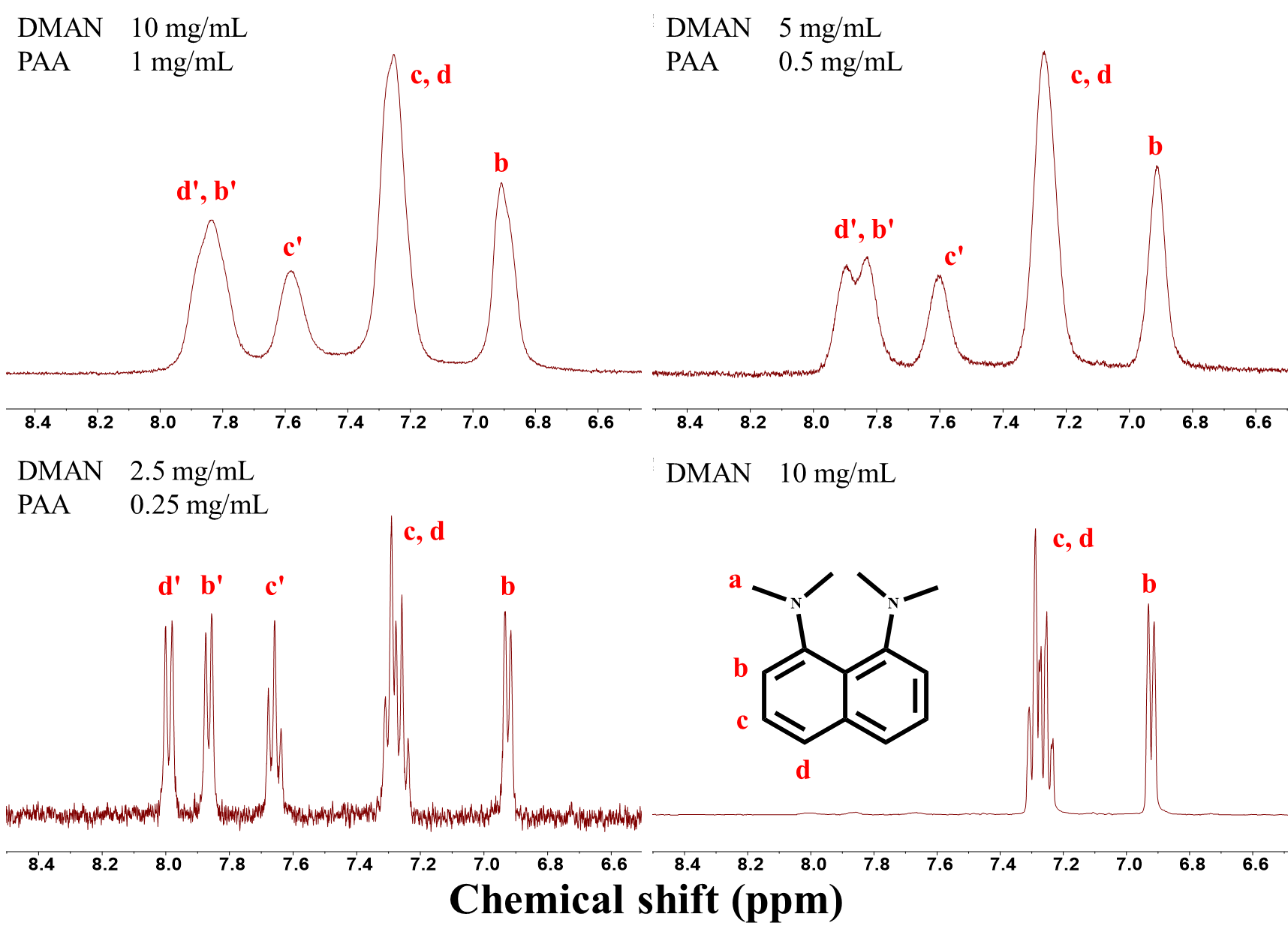


Figure S11. ^1^H NMR spectra of DMAN and DMAN-PAA with different concentration (chemical shift from 8.5 to 6.5 ppm).


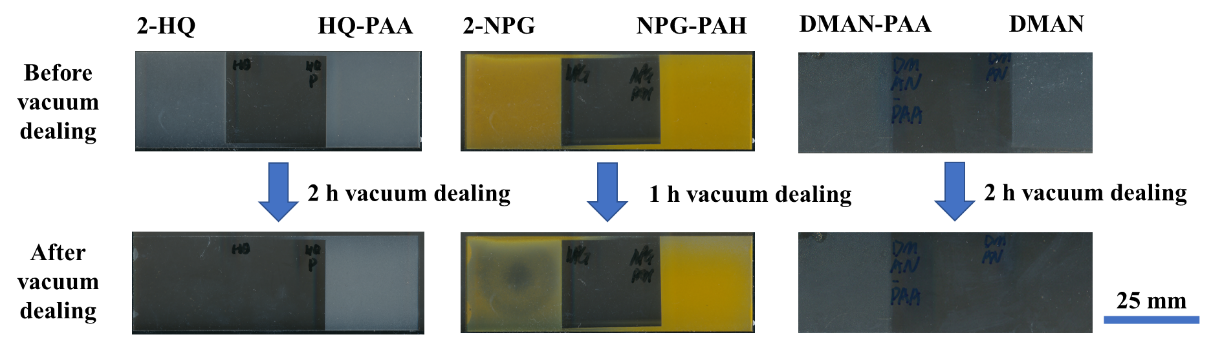


Figure S12. Optical images of 2-NPG, 2-HQ, NPG-PAH, and HQ-PAA before and after vacuum dealing.


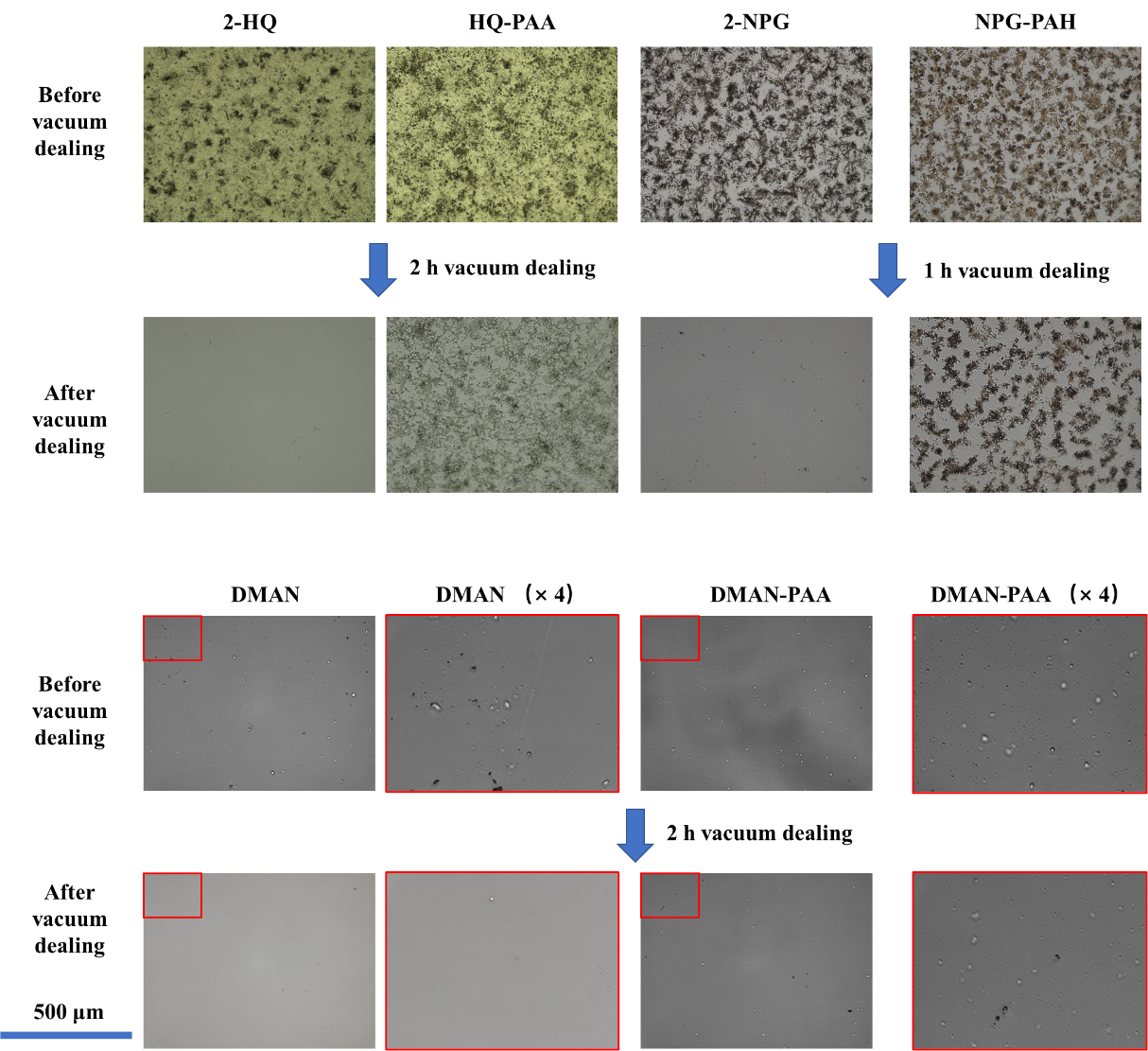


Figure S13. Optical microscope images of 2-NPG, 2-HQ, NPG-PAH, and HQ-PAA before and after vacuum dealing.

# MALDI MS spectra


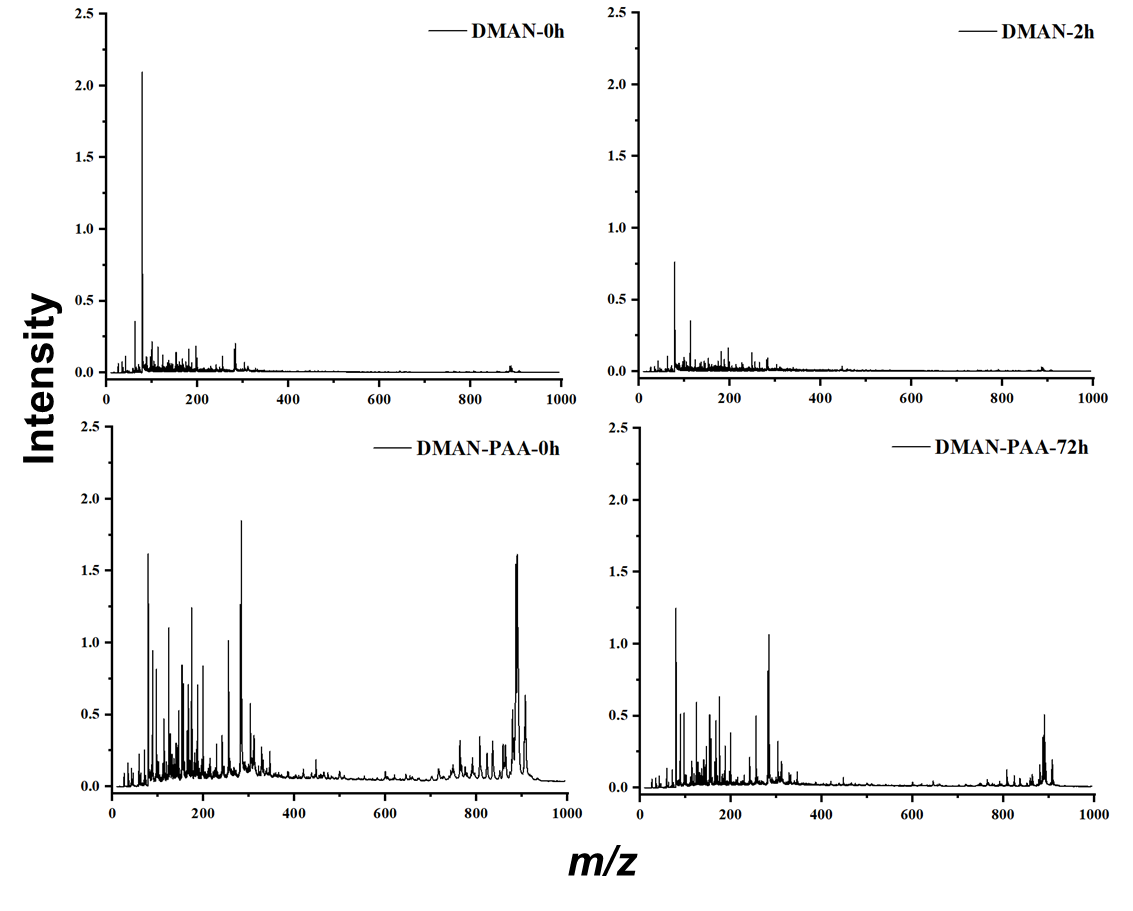


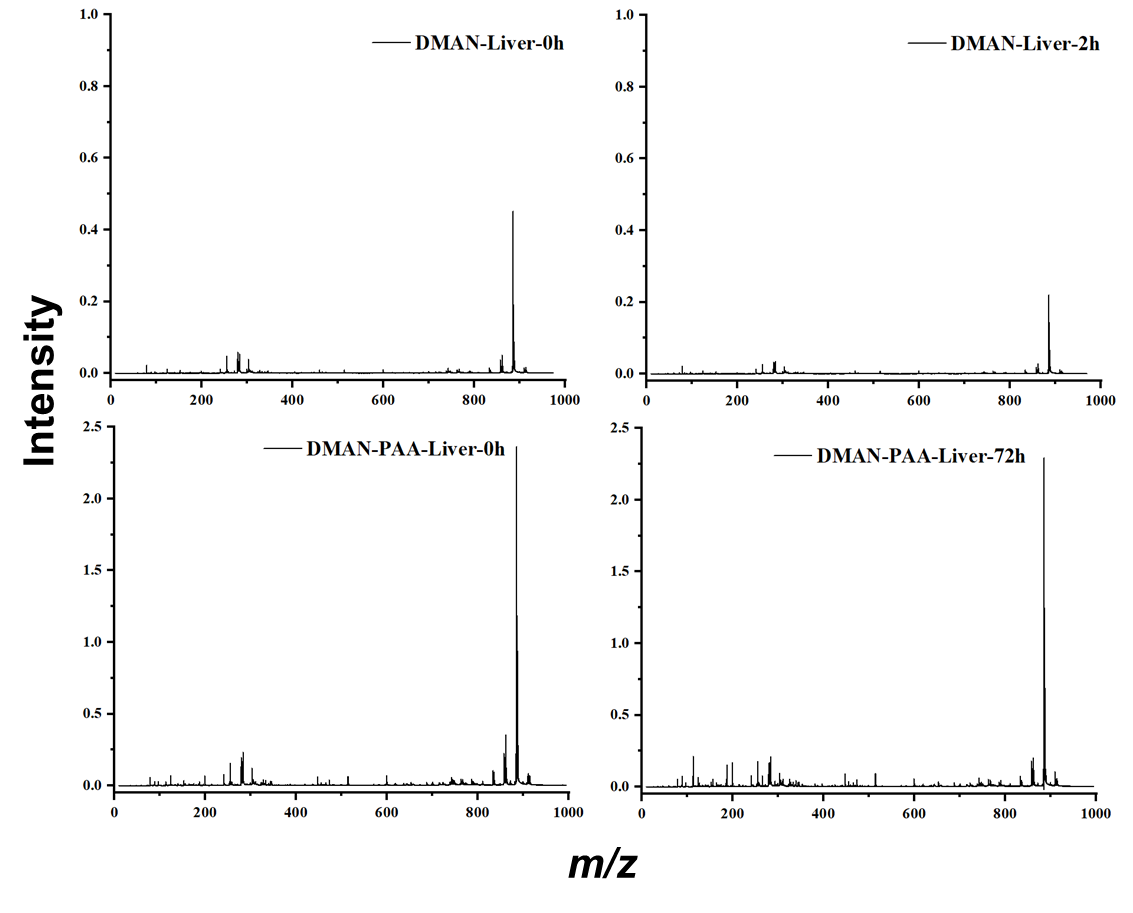


Figure S14. Mass spectra of MALDI MSI (DMAN and DMAN-PAA, Figure 1 and Figure 2).


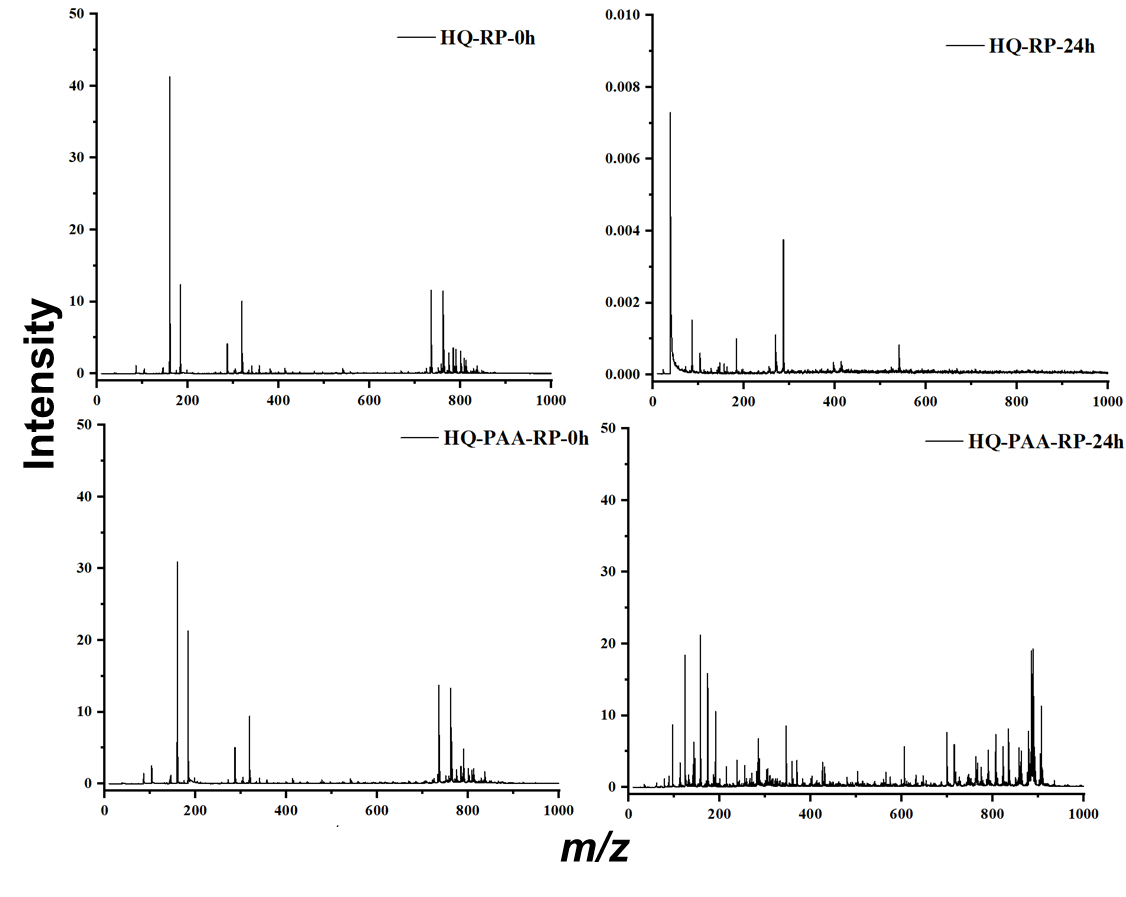


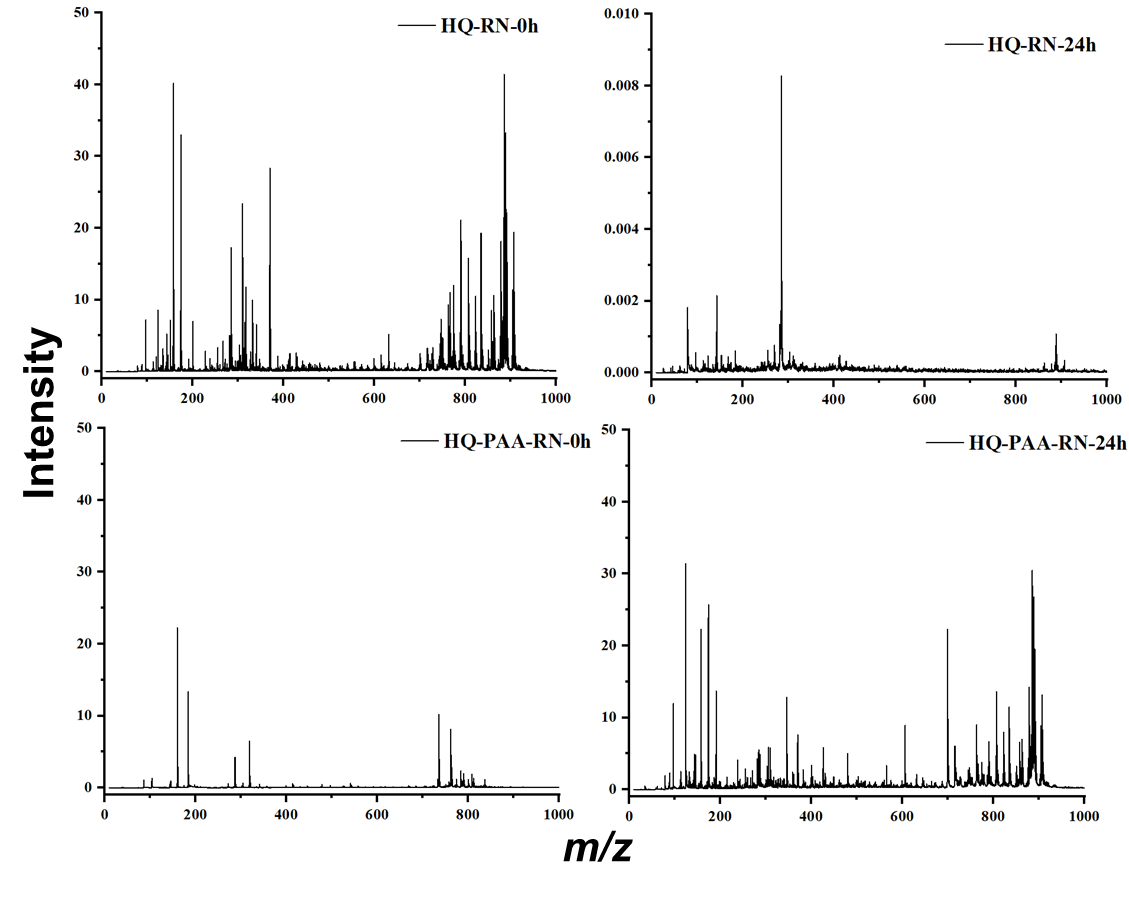


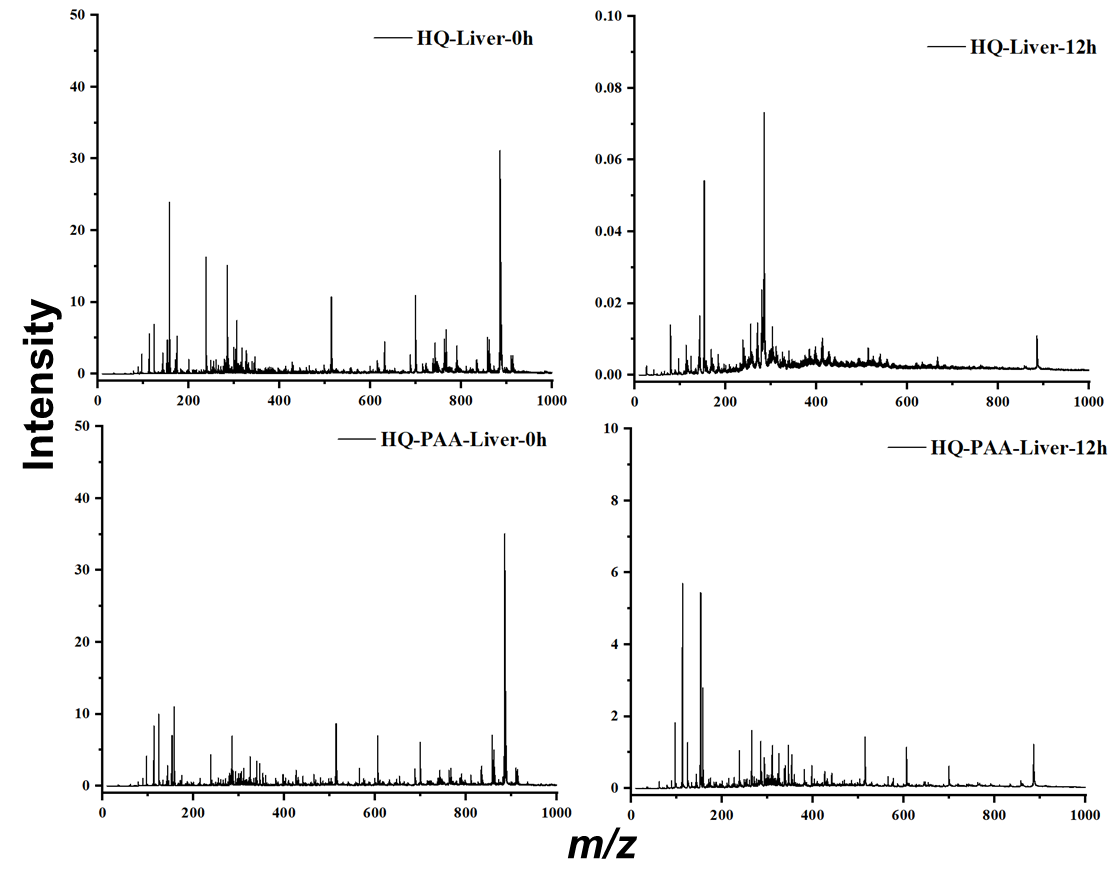


Figure S15. Mass spectra of MALDI MSI (2-HQ and HQ-PAA, Figure 5, Figure S22, and Figure S23).


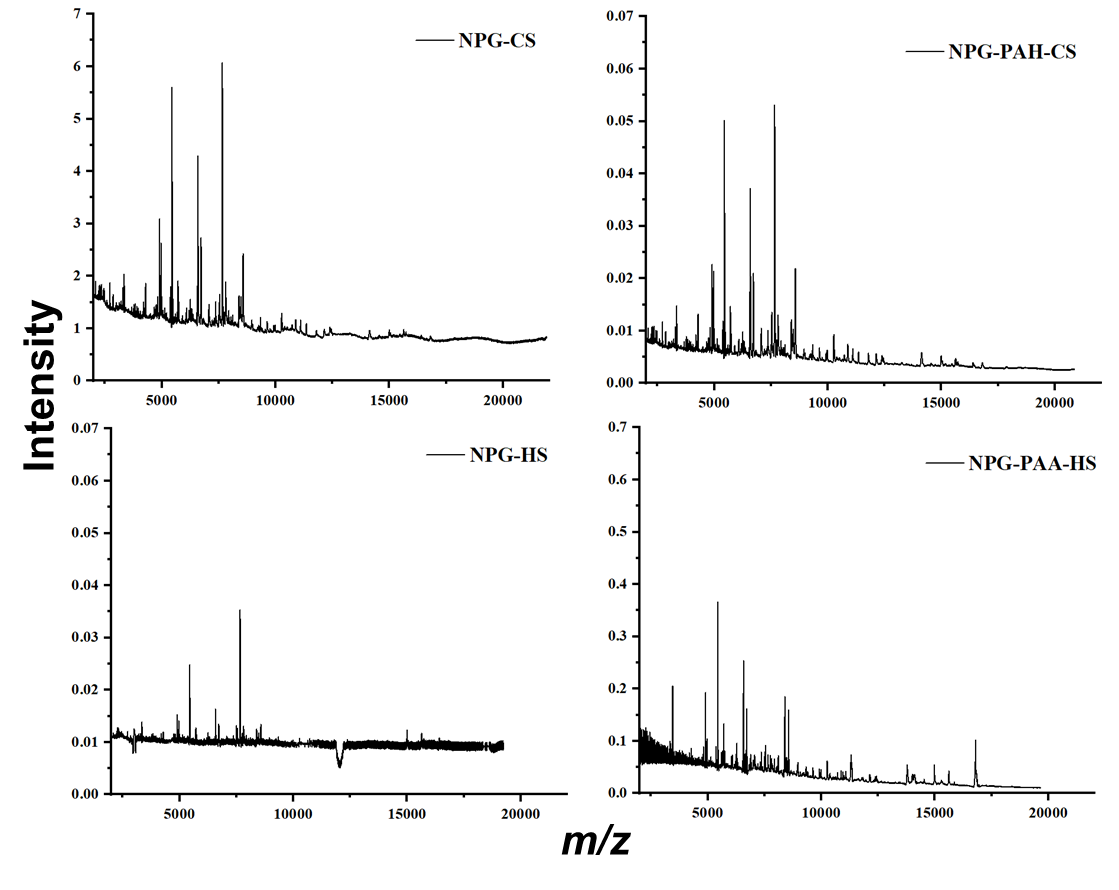


Figure S16. Mass spectra of MALDI MSI (2-NPG and NPG-PAH, Figure 4 and Figure S21, coronal section CS, horizontal section HS).


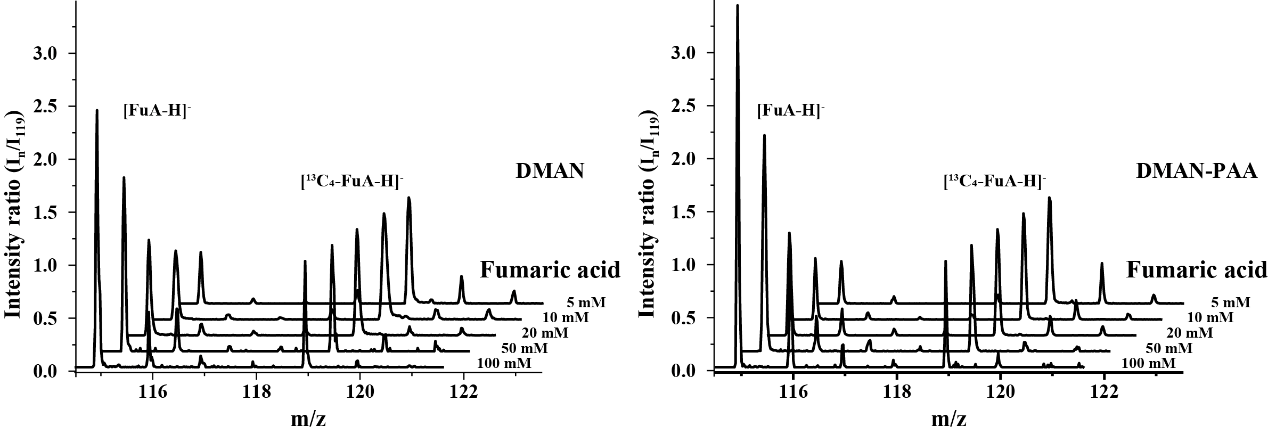


Figure S17. Mass spectra of calculable quantification for DMAN and DMAN-PAA assisted fumaric acid.


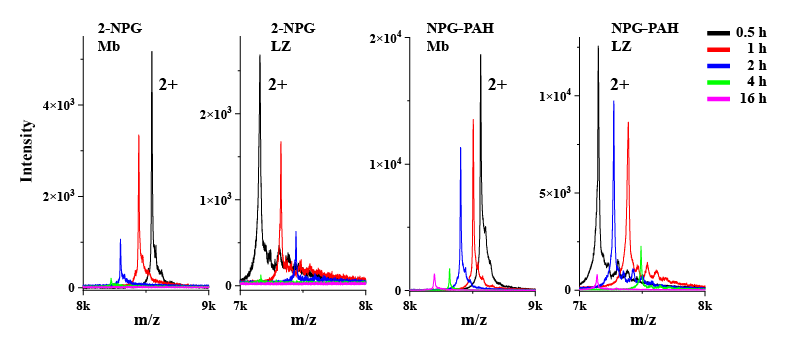


Figure S18. Mass spectra of 2-NPG and NPG-PAH assisted Mb and LZ with different vacuum dealing.


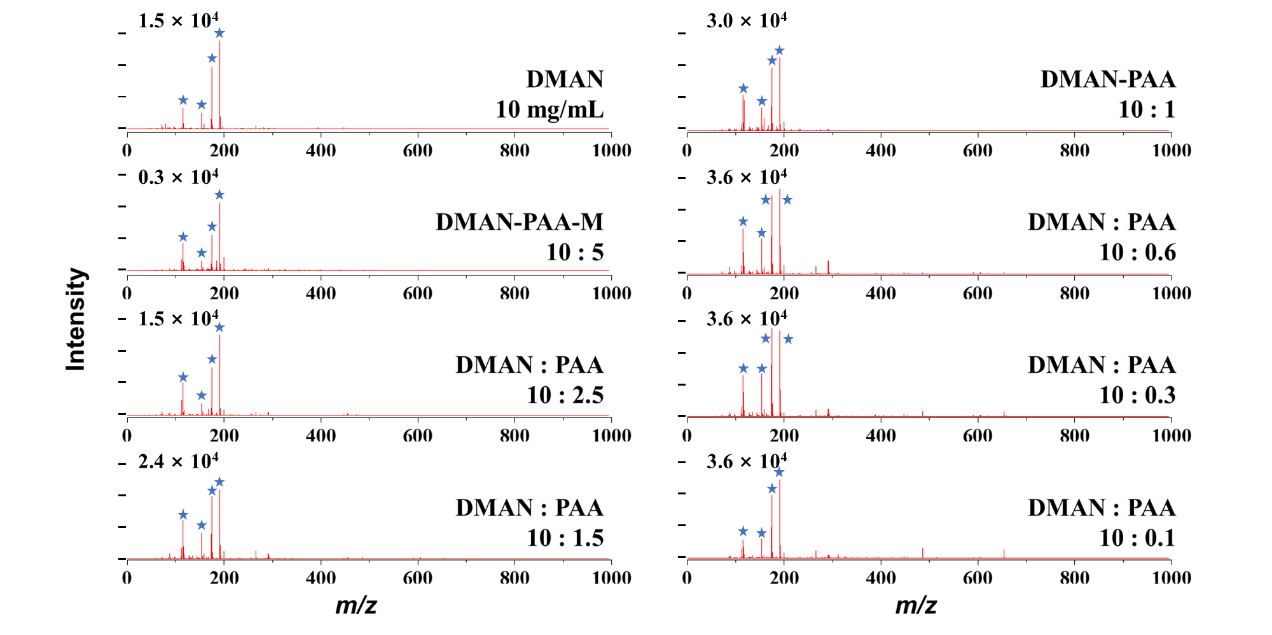


Figure S19. Mass spectra of DMAN and DMAN-PAA assisted four standard samples, His, AA, CA and FuA with different concentration of PAA (10 mg/mL DMAN and 5, 2.5, 1.5, 1, 0.6, 0.3, 0.1 mg/mL PAA).


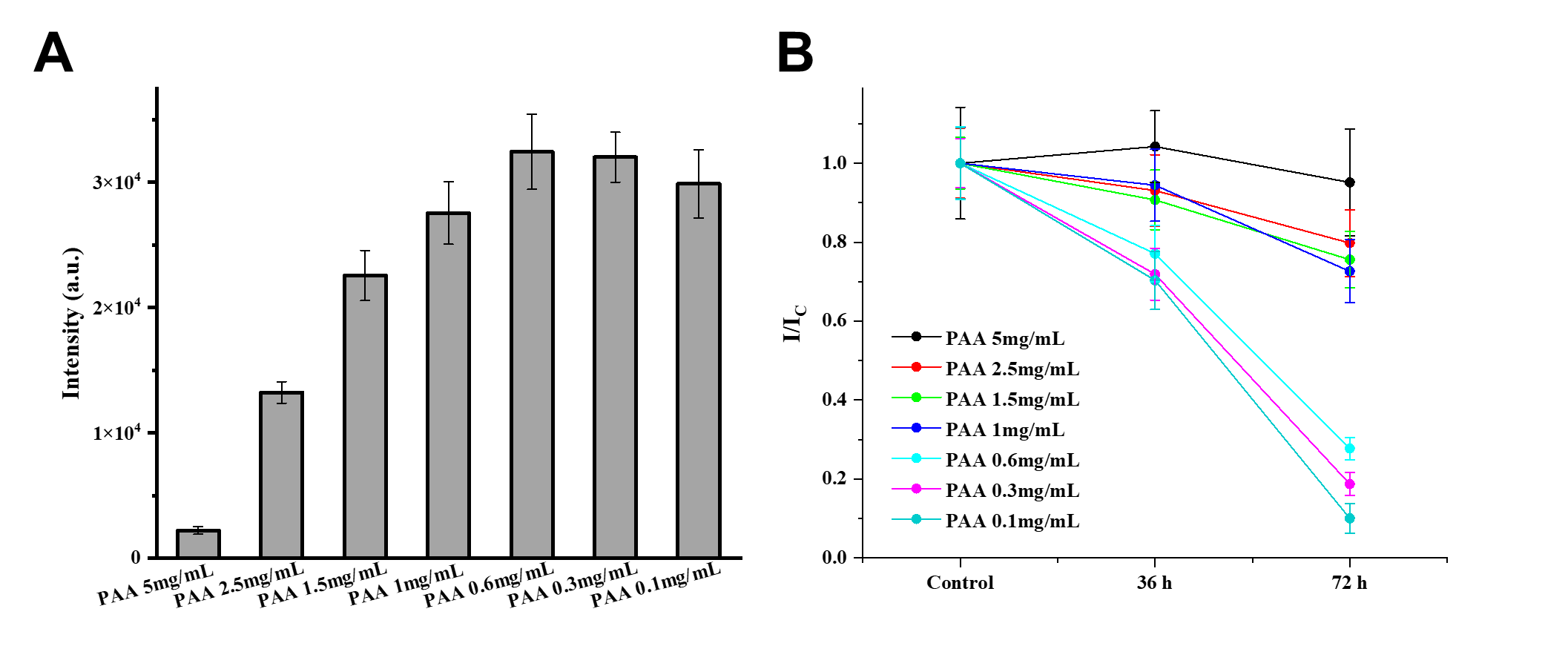


Figure S20. Changing ion signal intensity of DMAN-PAA assisted CA with a series of concentration of PAA before (A) and after vacuum dealing (B).

# MALDI MS imaging


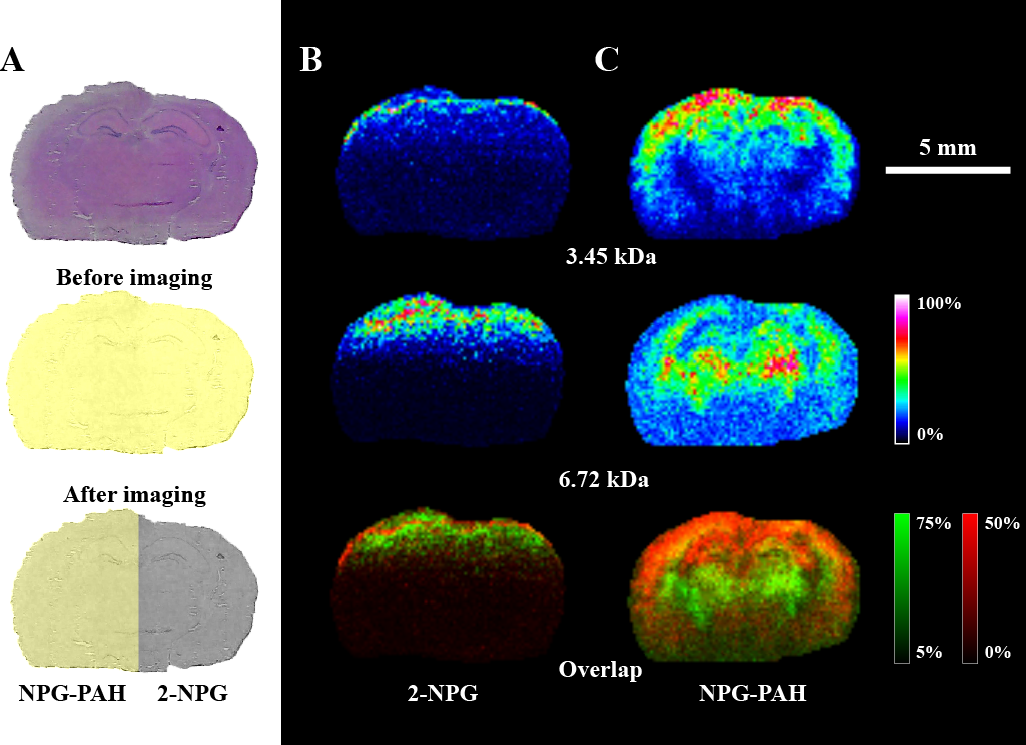


Figure S21. PAH enhancing 2-NPG vacuum stability improved mouse brain coronal section imaging. (A) H&E staining of mouse brain coronal section. MALDI MS imaging consequences of two individual proteins and the overlap with 2-NPG (B) and NPG-PAH (C) assisted. MS imaging experiments were performed in positive mode with laser energy adjusted to 60% and the special resolution set at 200 μm.


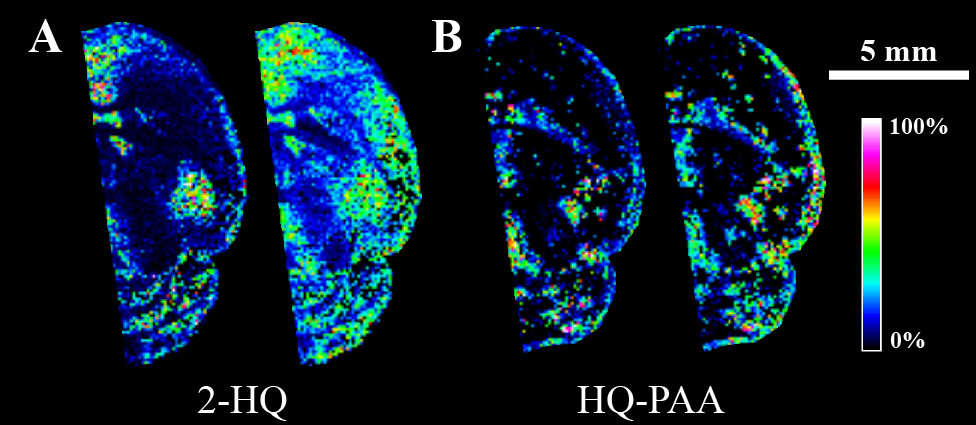


Figure S22. MALDI MS imaging consequences of 2-HQ (A) and HQ-PAA (B) assisted two features (left m/z 1484, right m/z 1514) with reflection positive (RP) mode. MS imaging experiments were performed in negative mode with laser energy adjusted to 55% and the special resolution set at 100 μm.


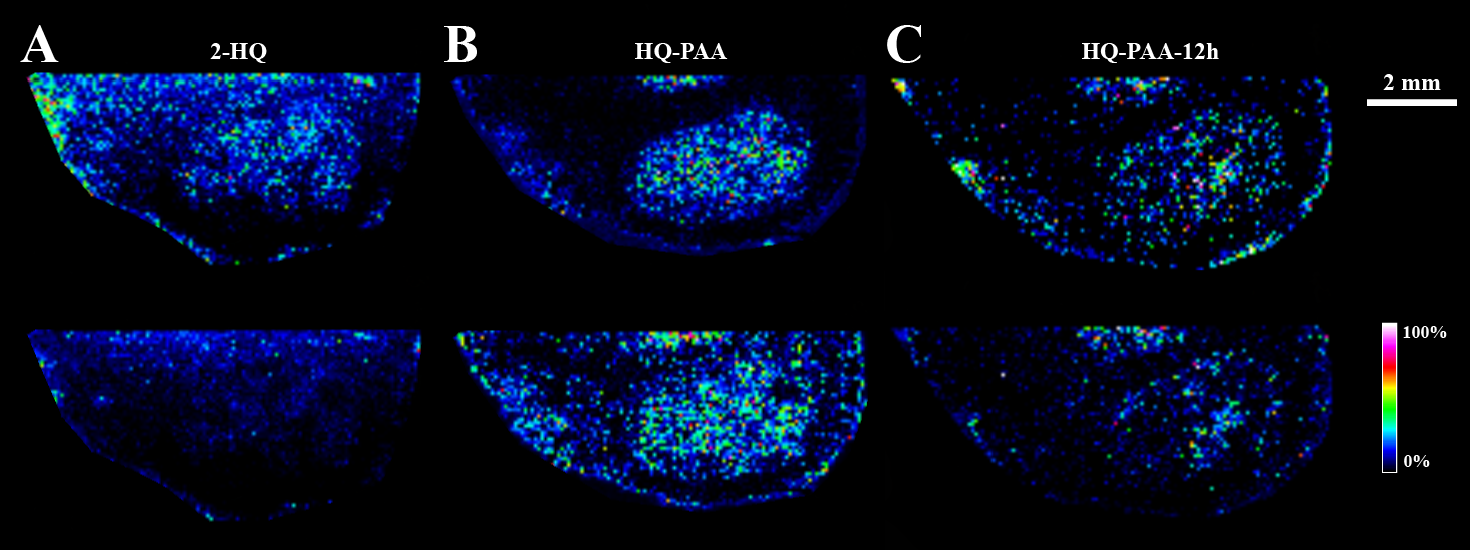


Figure S23. MALDI MS imaging consequences of PAA improving 2-HQ imaging property besides vacuum stability. Two extra features (top m/z 187, down m/z 606) with better distribution in HQ-PAA (B-C) assisted liver tissue imaging than HQ (A). MS imaging experiments were performed in negative mode with laser energy adjusted to 55% and the special resolution set at 50 μm.


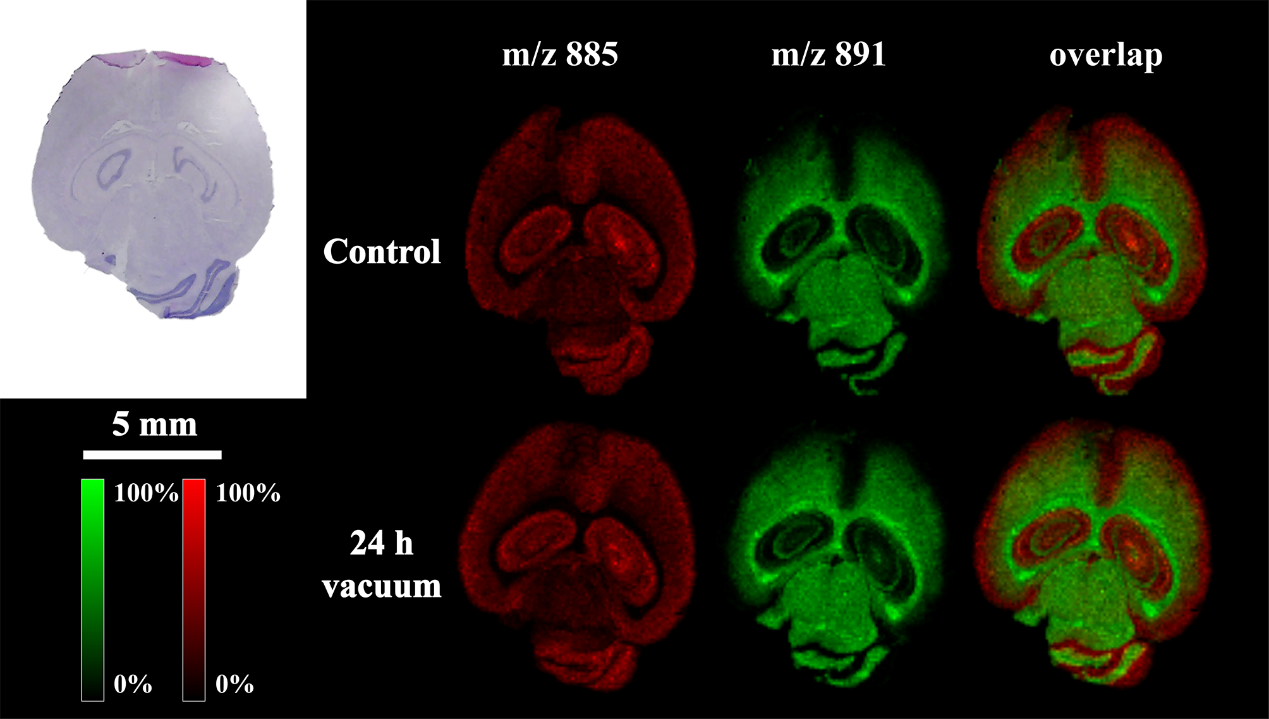


Figure S24. MALDI MS imaging consequences of PAA improving DMAN imaging property without and with 24 h vacuum dealing. MS imaging experiments were performed in negative mode with laser energy adjusted to 55% and the special resolution set at 100 μm.
